# Persistence of adoptively transferred T cells with a kinetically engineered IL-2 receptor agonist

**DOI:** 10.1038/s41467-019-12901-3

**Published:** 2020-01-31

**Authors:** Giulia Parisi, Justin D. Saco, Felix B. Salazar, Jennifer Tsoi, Paige Krystofinski, Cristina Puig-Saus, Ruixue Zhang, Jing Zhou, Gardenia C. Cheung-Lau, Alejandro J. Garcia, Catherine S. Grasso, Richard Tavaré, Siwen Hu-Lieskovan, Sean Mackay, Jonathan Zalevsky, Chantale Bernatchez, Adi Diab, Anna M. Wu, Begoña Comin-Anduix, Deborah Charych, Antoni Ribas

**Affiliations:** 1 Department of Medicine, David Geffen School of Medicine at UCLA, Los Angeles, CA USA; 2 Department of Medical and Molecular Pharmacology, David Geffen School of Medicine at UCLA, Los Angeles, CA USA; 3 Isoplexis Corporation, Branford, CT USA; 4 Department of Surgery, David Geffen School of Medicine at UCLA, Los Angeles, CA USA; 5 Parker Institute for Cancer Immunotherapy, San Francisco, CA USA; 6 Regeneron Pharmaceuticals, Inc., Tarrytown, NY USA; 7 Nektar Therapeutics, San Francisco, CA USA; 8 Department of Melanoma Medical Oncology, Division of Cancer Medicine, University of Texas MD Anderson Cancer Center, Houston, TX USA; 9 Department of Molecular Imaging and Therapy, Beckman Research Institute of the City of Hope, Duarte, CA USA; 10 Johnson Comprehensive Cancer Center, University of California, Los Angeles, CA USA; 11 Third Rock Ventures, San Francisco, CA USA

**Keywords:** Cancer immunotherapy, Interleukins

## Abstract

Interleukin-2 (IL-2) is a component of most protocols of adoptive cell transfer (ACT) therapy for cancer, but is limited by short exposure and high toxicities. NKTR-214 is a kinetically-engineered IL-2 receptor βγ (IL-2Rβγ)-biased agonist consisting of IL-2 conjugated to multiple releasable polyethylene glycol chains resulting in sustained signaling through IL-2Rβγ. We report that ACT supported by NKTR-214 increases the proliferation, homing and persistence of anti-tumor T cells compared to ACT with IL-2, resulting in superior antitumor activity in a B16-F10 murine melanoma model. The use of NKTR-214 increases the number of polyfunctional T cells in murine spleens and tumors compared to IL-2, and enhances the polyfunctionality of T and NK cells in the peripheral blood of patients receiving NKTR-214 in a phase 1 trial. In conclusion, NKTR-214 may have the potential to improve the antitumor activity of ACT in humans through increased in vivo expansion and polyfunctionality of the adoptively transferred T cells.

## Abstract (web summary)

Adoptive cell transfer (ACT) of T cells for tumor treatment often requires IL-2 administration. Here, the authors show that a modified IL-2 cytokine (NKTR-214) can outperform IL-2 in a melanoma mouse model.

## Introduction

Adoptive cell transfer (ACT) of T cells that recognize specific tumor antigens has achieved promising results in patients with advanced cancers^1^. Clinical protocols of ACT infusing ex vivo expanded tumor-infiltrating lymphocytes (TIL) or T cell receptor (TCR) engineered peripheral blood T cells require the concomitant administration of interleukin 2 (IL-2, aldesleukin), usually administered intravenously at the highest tolerated dose every 8 h in an inpatient hospital setting to support expansion and function of the adoptively transferred cells^2^. This dosing regimen results in a high peak of exposure explaining the acute toxicities, which is of very short duration and suboptimally activates the IL-2 receptor^3^. There have been attempts at decreasing the toxicity and improving the pharmacokinetics of IL-2 by lowering the doses and administering subcutaneously, but it is unclear if the benefit is maintained compared to high dose IL-2^4^. In addition, IL-2 has pleiotropic stimulatory effects by activating effector T cells and natural killers (NK) through the binding of the intermediate-affinity heterodimeric receptor IL-2Rβγ, but it also interacts with the high affinity heterotrimeric receptor complex IL-2Rαβγ promoting expansion of regulatory T cells (Tregs), which have known immune suppressive roles in the tumor microenvironment^5–7^. These limitations imposed by high dose IL-2 have restricted the use of ACT in the clinic^8^.

Different strategies have been pursued in order to develop gamma chain-signaling cytokines with an improved safety profile relative to the native IL-2 cytokine and that selectively expand effector T cells over Tregs^9–12^. NKTR-214 (also known as bempegaldesleukin) is a prodrug with the same amino acid sequence as the clinically approved IL-2, but which is conjugated with multiple releasable chains of polyethylene glycol (PEG). In its prodrug form, NKTR-214 does not bind to IL-2 receptors and does not activate T cell signaling. However, when exposed to physiological conditions, the PEG chains sequentially release over time, allowing the cytokine to bind and trigger signaling through the IL-2 receptor complex. In its most active forms, 1-PEG-IL-2 and 2-PEG-IL-2, the position of the PEGylation reduces the interaction between IL-2 and its cognate IL-2Rα subunit, while the affinity to the IL-2Rβγ complex remains relatively unchanged^3,13^. Prior studies have provided evidence that the preferential activation of IL-2Rβγ over IL-2Rα results in an increased ratio of tumor-killing CD8 T cells to Treg in NKTR-214 treated mice compared with IL-2 (400 versus 18 fold, respectively)^3,13^. Free unconjugated-IL-2 is essentially undetectable as it clears faster than it forms. NKTR-214 as a monotherapy and in combination with anti-PD-1 antibodies or Toll-like receptors (TLR) agonists, is currently being evaluated in an outpatient setting in several clinical trials, showing early evidence of efficacy in multiple tumor types along with a favorable outpatient dosing^14^.

In the current work, we test the hypothesis that co-administered NKTR-214 can outperform IL-2 to improve the in vivo expansion, activation, and persistence of adoptively transferred effector T cells to improve anti-tumor efficacy. To test this principle, we used the pre-clinical pmel-1 mouse model to study the behavior of tumor-specific CD8 T cell expansion when NKTR-214 is combined with ACT compared to ACT + IL-2. All CD8 T cells from pmel-1 mice have a transgenic T cell receptor (TCR) specific for the melanoma antigen gp100 expressed by B16-F10 murine melanoma cells. This model allows controlled evaluation of the anti-tumor activity, biodistribution, in vivo functionality, and gene expression profile from these T cells when combined with either NKTR-214 or IL-2. In addition, we analyze the T cell polyfunctionality of murine T cells and also explore the polyfunctionality of human T cells obtained from patients treated with NKTR-214 within a single agent, phase I clinical trial.

## Results

### ACT + NKTR-214 achieves long-lasting anti-tumor responses

**Fig. 1:**
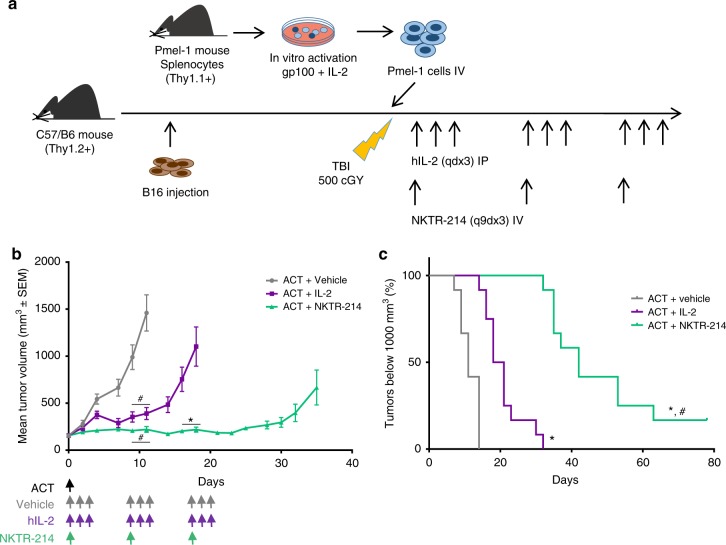
Superior antitumor response by adoptively transferred T cells with NKTR-214 compared to IL-2. **a** Schematic of the pmel-1 ACT model. Time points in days throughout the text refer to the number of days post-administration of ACT + NKTR-214 or ACT + IL-2 at day 0. Subsequent to day 0, no further ACT was given. A second dose of either NKTR-214 (one dose) or IL-2 (qdx3) was given at day 9 and day 18 per the q9dx3 dosing regimen. **b** Changes in mean tumor volume over time. Data are means ± s.e.m (*n* = 12). *p < 0.0001 compared to ACT + IL-2; ^#^*p* < 0.0001 compared to vehicle (two-way ANOVA, with Bonferroni-Dunn multiple comparisons test). (**c**)Tumor growth delay was assessed for each tumor growth curve by interpolating the time to achieve tumor volume of 1000 mm^3^. ^#^*p* < 0.0001 compared to ACT + IL-2 (Log-Rank Mantel–Cox test). The experiment is representative of three replicates.

The anti-tumor effects of NKTR-214 to support the expansion and functionality of adoptively transferred antitumor T cells was evaluated in mice bearing established B16-F10 murine melanoma tumors larger than 150 mm^3^ at the time of ACT. C57BL/6 mice were implanted with B16-F10 and received pmel-1 Thy1.1+ T lymphocytes activated in vitro with gp100 peptide followed by systemic treatment with either IL-2 or NKTR-214 after a lymphodepletion regimen using 500 cGy total body irradiation (Fig. 1a). A dose escalation was performed to define the dose of NKTR-214 to administer with ACT in order to achieve an anti-tumor response with acceptable toxicity profile in mice (Supplementary Fig. 1a–d). NKTR-214 in combination with ACT was well-tolerated up to 0.8 mg/kg of NKTR-214, which was given once every 9 days for three doses (q9dx3) as previously shown^3^. No difference in tumor delay was observed with a fourth dose (Supplementary Fig. 1e). The previously reported dosing regimen for ACT + IL-2 was used as a comparator (0.4 mg/kg for three consecutive days, qdx3)^15^. In order to reasonably balance the different pharmacokinetic profiles between NKTR-214 and IL-2, we repeated the qdx3 IL-2 dosing regimen every 9 days to coincide with the q9dx3 schedule for NKTR-214. The adoptively transferred cells were only administered on day 1 after lymphodepletion, and ACT was not repeated with subsequent administrations of either NKTR-214 or IL-2.

ACT + NKTR-214 led to significant tumor growth inhibition with less frequent dosing in the aggressive B16-F10 model compared to ACT + IL-2 or ACT + vehicle (Fig. 1b, c). As demonstrated previously^16^, pmel-1 ACT without IL-2 supplementation had little to no antitumor benefit in this model, highlighting the critical role of IL-2 to provide robust antitumor activity with ACT. Mice receiving ACT + NKTR-214 showed a median tumor growth delay (evaluated as time to reach a tumor volume of 1000 mm^3^) of 42 days compared to ACT + IL-2 or ACT + vehicle (19.5 and 11 days, respectively, Fig. 1c). Two out of 12 mice in the ACT + NKTR-214 had small tumors neither growing nor shrinking until the end of the study (Fig. 1c and Supplementary Fig. 2a). Tumors from these mice showing long term response were harvested and processed for immunohistochemical (IHC) analysis, which revealed persistent tumor-infiltrating CD8 T cells in the samples (Supplementary Fig. 2b). To monitor potential treatment toxicities, we observed mice for any appearance of clinical signs and measured their body weight throughout the course of the experiments. Results showed that mice did not present any weight changes or other observable side effects after injection of 0.8 mg/kg of NKTR-214 in combination with ACT (Supplementary Fig. 3a). There was a trend towards increased CD8 T cells infiltrates in liver and kidney on day 5 after ACT + NKTR-214 or ACT + IL-2 as assessed using IHC staining. These ranged from 0–6% positive cells in kidney and 0–40% in liver, declining to less than 10% by day 9, prior to administration of the second dose. These effects did not result in obvious clinical signs based on the activity level and observed behavior of the mice (Supplementary Fig. 3b, c).

### NKTR-214 expands adoptively transferred CD8 T cells

**Fig. 2:**
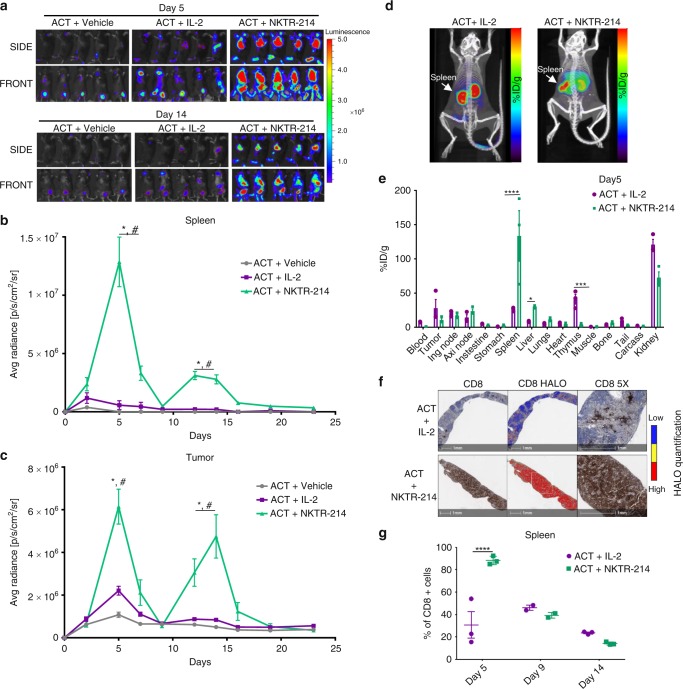
Increased T cell expansion in the spleen and durable tumor infiltration with NKTR-214 treatment. **a** Time-course bioluminescence imaging (BLI) of firefly luciferase-labeled pmel-1 T cell trafficking in vivo. Representative images on days 5 and 14, five replicate mice per group. T cell expansion in spleen (upper panels, SIDE); mobilization and persistence in tumor (lower panels, FRONT). Scale bar shows the radiance, going from a minimum of 3.0 × 10^5^ e to a maximum of 5.0 × 10^6^ photons second^−1^ cm^−2^ steradian^−1^. **b**, **c** Quantitative imaging analysis of region of interest (ROI) of spleen (**b**) and tumor (**c**) through day 23 after ACT of pmel-1 T cells expressing luciferase. Mice were treated with the combination of ACT and NKTR-214 (0.8 mg/kg, q9dx3, i.v.) or with IL-2 (0.4 mg/kg, qdX3 every 9 days for 3 cycles, i.p.), or vehicle control. Signal in spleen reached the peak at day 5 for ACT + NKTR-214 compared to ACT + IL-2. *p < 0.0001 ACT + NKTR-214 compared to ACT + IL-2, ^#^*p* < 0.0001 compared to ACT + vehicle, two-way ANOVA with Tukey multiple comparisons test, *n* = 5, mean ± SEM. **d** Representative immuno-PET/CT images acquired on day 5 after treatment with ACT + IL-2 or ACT + NKTR-214 (*n* = 3/group) using Zr-89-labeled anti-CD8 cys-diabodies (cDb). Scale bar represents the percent-injected dose per gram of tissue (%ID/g) detected, from low (black) to high (red) intensity. **e** Ex vivo analysis of T cell biodistribution in organs harvested at 24 h post-injection of Zr-89 cDb. %ID/g: injected dose per gram. The diabody and residualizing radionucleotide undergo renal clearance, hence the high non-specific signal from kidneys. Mean ± s.e.m (*n* = 3),*p < 0.05, ***p < 0.001, ****p < 0.0001, unpaired *t*-test. **f** CD8+ staining of FFPE from spleen at day 5, representative image of three replicates per group. HALO software analysis showing intensity of marker expression, from low (blue) to high (red) expression level. **g** Digital pathology quantification of IHC slides using HALO software showing the percentage of CD8 T cell expansion in the spleen at different time points. ****p < 0.0001, mean ± s.e.m, unpaired *t*-test.

To better understand the mechanisms of improved antitumor activity and T cell trafficking with the ACT + NKTR-214 treatment, pmel-1 Thy1.1 + T cells were genetically modified to express firefly luciferase, which allows detection of T cells in vivo using bioluminescence imaging (BLI) to follow the biodistribution of the adoptively transferred T-cells and tumor-specific homing (Fig. 2a–c). BLI analysis showed that ACT without IL-2 resulted in minimal to no in vivo expansion of the adoptively transferred pmel-1 cells but there was some accumulation in the tumors due to cognate antigen recognition (Fig. 2a, left column), as we have previously described^17^, further portraying the key role of IL-2 supplementation for an effective antitumor response with ACT. BLI images from the ACT + NKTR-214 treatment greatly increased T cell expansion in the spleen from day 5 to day 7 compared to ACT + IL-2 (Fig. 2a, middle and right columns). At day 5, quantification of BLI in serial images with region of interest (ROI) analysis at the site of spleen revealed an average radiance 14-fold higher in ACT + NKTR-214 compared to ACT + IL-2 treated mice (Fig. 2b). These data were supported by immuno-PET imaging using cys-diabody (cDb) targeting CD8 in vivo, allowing whole body non-invasive imaging to study the sites of accumulation of CD8 T cells (Fig. 2d). In addition, ex vivo biodistribution analysis of radioactivity from harvested organs demonstrated that the signal in the spleen was 5-fold higher in the ACT + NKTR-214 group compared with the ACT + IL-2 group on day 5 after treatment (Fig. 2e). CD8 IHC of spleens analyzed by sequential digital pathology confirmed a significantly higher expansion of CD8 T cells in mice treated with ACT + NKTR-214 compared to ACT + IL-2 (Fig. 2f, g). NKTR-214 triggered a rapid reconstitution of splenic immune cells after lymphodepletion in the ACT + NKTR-214 treated mice, resulting in splenocyte cell counts seven times higher than ACT + IL-2-treated mice 6 days after total body irradiation (Supplementary Fig. 4). It was noted that by day 9 and before the second dose of NKTR-214 or IL-2, a substantial decrease in the BLI signal was observed in spleen and tumor, independently of the treatment (Fig. 2b, c and Supplementary Fig. 5a), consistent with a q9d dosing schedule. The use of anti-CD8 immuno-PET once again provided confirmation of these kinetics by observation of a temporary decrease in the CD8 T cell signal in spleen and tumor by day 9 (Supplementary Fig. 5b, c). Strikingly, the second NKTR-214 dose administered on day 9 triggered a new expansion of the adoptively transferred T cells (given only on day 1) in spleen and tumor, which persisted from day 12 to day 17. In sharp contrast, minimal secondary expansion was noted in the group re-dosed with IL-2 (Fig. 2a–c). The peak of T cell infiltration in the tumor after the second NKTR-214 dose was reached at day 14, with an average radiance 6-fold higher in the ACT + NKTR-214 group compared to the ACT + IL-2 group (Fig. 2a–c). No further expansion of CD8 T cells was observed after the third dose of NKTR-214 performed at day 18 (Fig. 2b, c).

### Characterization of cell subsets modulated by ACT + NKTR-214

**Fig. 3:**
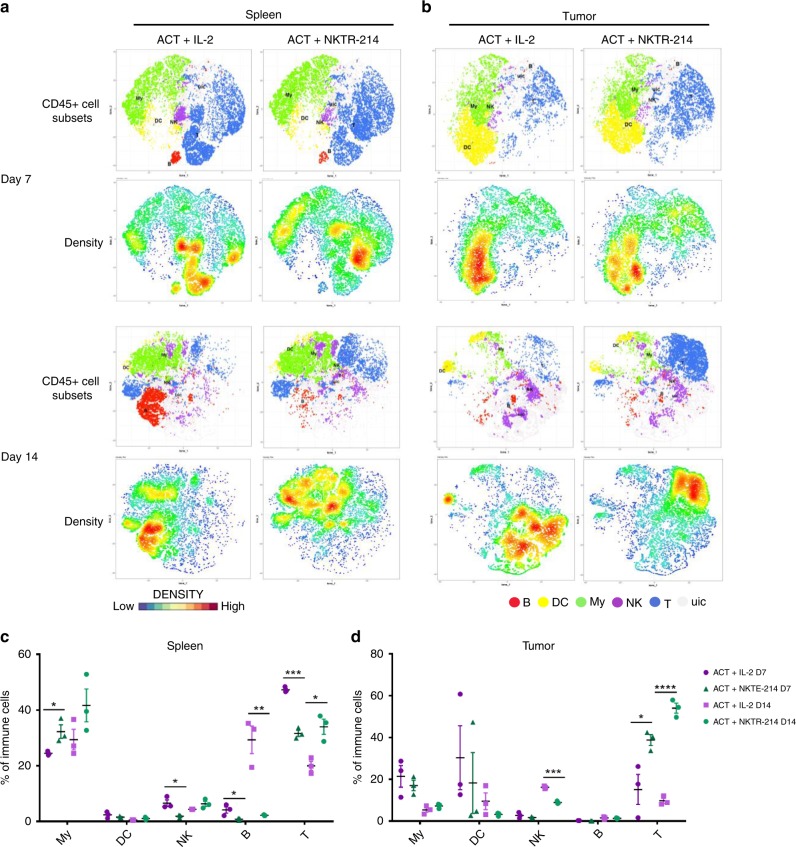
Identification of differentially activated immune cells A. **a**, **b** Mass cytometry at day 7 and 14 after treatment with t-SNE plots showing annotated clusters (upper panels) and density plots (lower panels) in spleen (**a**) and tumor in mice treated with CT + NKTR-214 compared to ACT + IL-2. **b** Density scale bar represents marker expression of cells for a given cluster, ranging from low expression (blue) to high expression (red). In the CD45+ cell subsets panels, red dots represent B cells (B), yellow dots: dendritic cells (DC), green dots: myeloid cells (My), purple dots: natural killer cells (NK), blue dots: T cells (T), light gray dots: unidentified cluster (uic). **c**, **d** Cell frequencies were calculated for the annotated clusters and displayed on a per-mouse basis in spleen (**c**) and tumor (**d**). Mean ± s.e.m (*n* = 3), *p < 0.05, **p < 0.01, ***p < 0.001, ****p < 0.0001, unpaired *t*-test. The experiment is representative of two replicates.

**Fig. 4:**
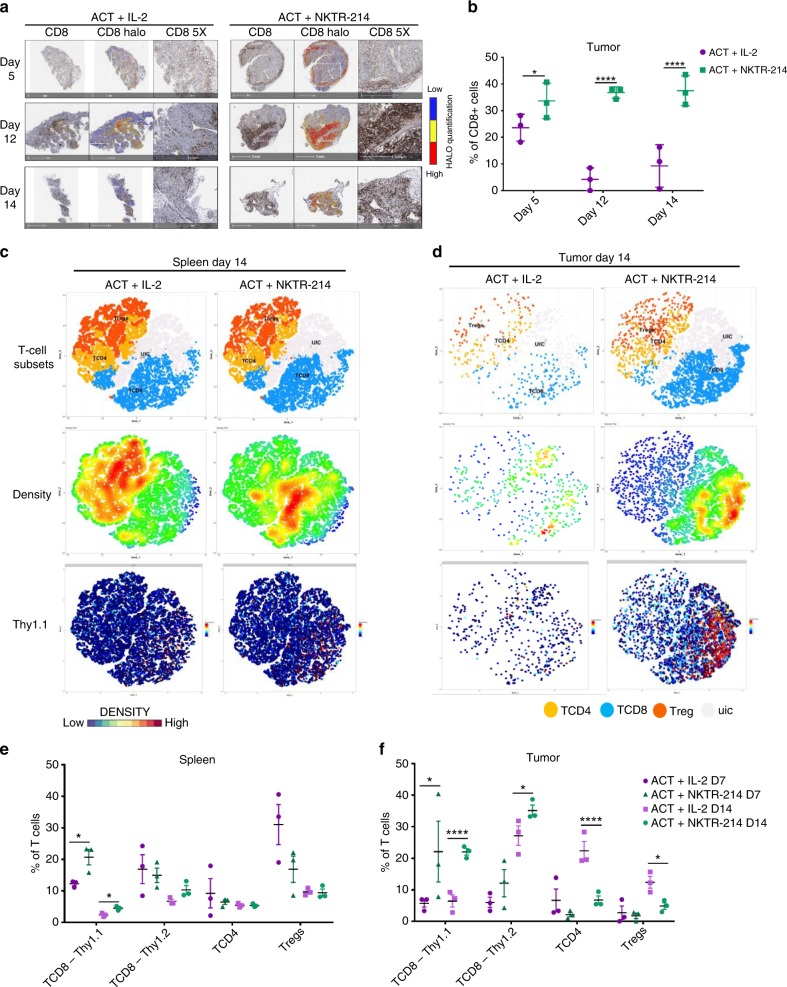
Increased T cell infiltration is a significant component of NKTR-214′s anti-tumor efficacy. **a** Immunohistochemistry (IHC) CD8 T cells staining in tumors at different time points, representative image of three replicates per group. HALO software analysis showing intensity of marker expression, from low expression (blue) to high expression (red). **b** HALO software digital quantification of IHC slides showing the percentage of CD8 T cells infiltrating the tumor at different time points. *p < 0.05, ****p < 0.0001, mean ± s.e.m, unpaired *t*-test. **c**, **d** Mass cytometry results on T cells compartment. T-SNE plots showing annotated clusters (upper panels), density plots (middle panels) and Thy1.1 marker expression level plot (lower panels) in spleen (**c**) and tumor at day 14 (**d**). Density scale bar represents marker expression of cells for a given cluster, ranging from low expression (blue) to high expression (red). In the T cell subsets panels, yellow dots represent CD4+ T cells (TCD4), blue dot: CD8+ T cells (TCD8), orange dots regulatory T cells (Treg), light grey dots: unidentified cluster (uic). **e**, **f** Cell frequencies at day 7 and 14 after treatment were calculated for the annotated T cell clusters and displayed on a per-mouse basis in spleen (**e**) and tumor (**f**). Mean ± s.e.m (*n* = 3), *p < 0.05, ****p < 0.0001, unpaired *t*-test. The experiment is representative of two replicates.

To further characterize the functional phenotype of the treatment-induced immune cell populations, we performed mass cytometry in spleen and tumors of mice treated with ACT in combination with NKTR-214 or IL-2 at day 7 and 14, the peak of the first and second CD8 T cell expansion. Our panel comprised of T, NK, B, and myeloid cell lineage markers (Supplementary Table 1). We first manually annotated seven main immune populations from CD45+ cells (Supplementary Fig. 6a) using *t*-stochastic neighbor embedding (t-SNE)-generated heat maps corresponding to myeloid cells, dendritic cells (DC), NK cells, B cells and T cells (Fig. 3a, b). As expected, the clusters showing greater differences between treatments were T cell populations, both in spleen and tumor (Fig. 3a–d). Additionally, in the spleen the myeloid compartment was increased at day 7 and 14 by ACT + NKTR-214 treatment (Fig. 3a–c). The robust re-treatment effect was particularly striking after NKTR-214 compared to IL-2 at day 14 (5 days post-administration of the second dose), confirming the in vivo imaging result of Fig. 2c. The NK cluster expanded preferentially with ACT + IL-2 at day 7, and with ACT + NKTR-214 at day 14 (Fig. 3a–c). T-SNE density plots showed that IL-2 led to preferential B cells expansion in spleen, on days 7 and 14 (Fig. 3a). Interestingly, dendritic cell populations represented an important fraction of the tumor immune cells in both ACT + IL-2 and ACT + NKTR-214-treated mice at an early time point (30.3% and 18.2%, respectively, Fig. 3b–d). In the tumor at day 7, the two treatments did not differ significantly in the expansion of any immune population, except for T cells (Fig. 3b–d). At day 14, IL-2 preferentially expanded NK, while NKTR-214 expanded dramatically the T cell compartment, suggesting that the T cells are the main contributors in the enhanced anti-tumor response by NKTR-214 in this model. This finding was confirmed by IHC-based quantification of CD8-stained tumor sections, showing a significantly higher number of intratumor lymphocytes in the ACT + NKTR-214 group compared to ACT + IL-2 group at day 14 (37.5% vs 9.3% of total tumor cells, Fig. 4a, b).

### NKTR-214 favors CD8 T cell tumor infiltration over Tregs

We performed further T cell subset analyses using the mass cytometry data (Supplementary Fig. 6a). We identified three main subpopulations: CD8 T cells, CD4 T cells, and Tregs. Confirming what was described in the previous experiments, analysis of T cell populations revealed increased expansion and persistence of adoptively transferred Thy1.1+ CD8 T cells in spleen and tumor of mice treated with ACT + NKTR-214 compared to ACT + IL-2, both at days 7 and 14 days (Fig. 4c, d, Supplementary Fig. 6b, c). Density plots of the T cell clusters showed the preferential activation of tumor-specific Thy1.1 +CD8 T cells over Tregs in spleen and tumor for the ACT + NKTR-214-treated mice, while the opposite trend was observed in the ACT + IL-2 group, both at day 7 and 14 (Fig. 4c–f, Supplementary Fig. 6b, c). While the Thy1.1/Tregs ratio was two-fold higher in spleen after ACT + NKTR-214 compared to ACT + IL-2 at day 7 and 14 (Supplementary Fig. 7a), this ratio was 4 and 11-fold higher in tumor, respectively (Supplementary Fig. 7b). Remarkably, up to 40% of intratumor lymphocytes were tumor antigen-specific at day 7 and up to 22% at day 14 in the ACT + NKTR-214 group (Fig. 4f). Interestingly, NKTR-214 preferentially expanded Thy1.1+ tumor-specific CD8 T cells over the endogenous Thy1.2+ CD8 T cells in spleen at day 7 after treatment (Fig. 4e, Supplementary Figs. 6b and 7e). Ki-67 expression marker analysis showed that ACT + NKTR-214 treatment resulted in an increased proliferation of T cell clusters, particularly CD8 T cells in spleen and tumor at both time points (Supplementary Fig. 7c–e). The expression of PD-1 was low or absent in the spleen of both treatments (Supplementary Fig. 7c), while NKTR-214 enhanced activation of T cells in tumor in the mice treated with ACT + NKTR-214 (Supplementary Fig. 7d).

### NKTR-214 treatment increases immune-related gene expression

**Fig. 5:**
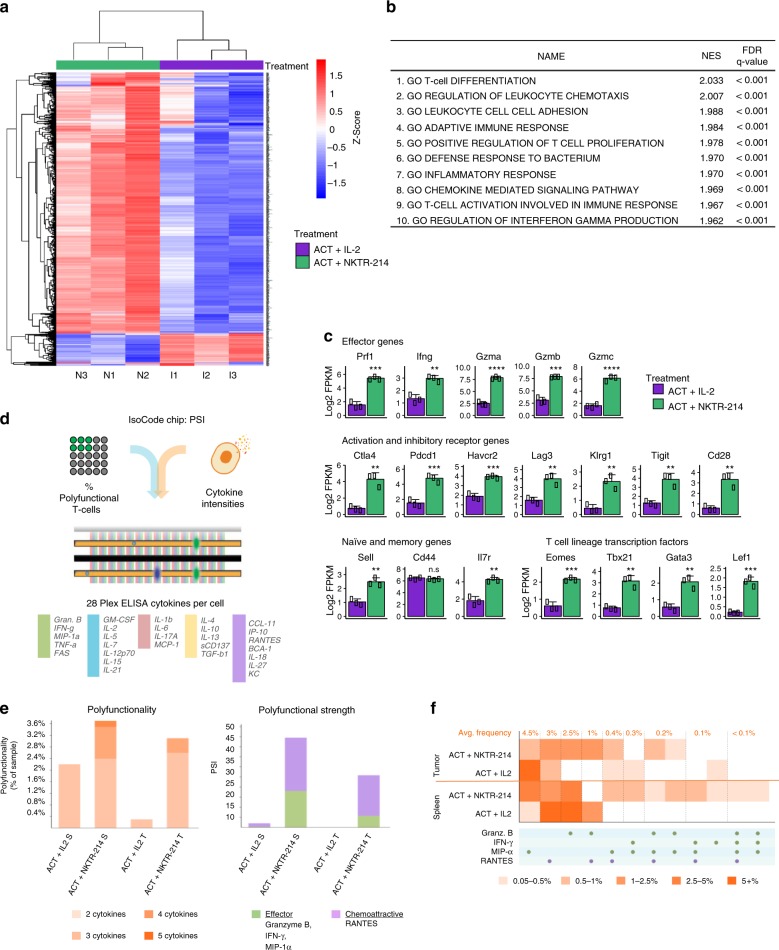
NKTR-214 elicits a marked upregulation of immune gene expression and T cell polyfunctionality. **a** Heatmap representing hierarchical clustering of the most variable genes (~1500 genes) in the ACT + NKTR-214 and ACT + IL-2 treated group (*n* = 3/group). **b** Gene set enrichment analysis of log2 fold-change of ACT + NKTR-214 and ACT + IL-2 samples, representing the top-10 biological pathways with significant gene induction. NES: normalized enrichment score; FDR: false discovery rate. **c** Bar charts of select T cell genes. Mean ± s.e.m (*n* = 3), **p < 0.01, ***p < 0.001, ****p < 0.0001, ns = not significant, unpaired *t*-test. **d**–**f** Single cell measure of polyfunctionality and polyfunctional strength of adoptively transferred T cells from spleen and tumors (pool of 3 each/group). **d** Schematic of the single cell IsoCode chip to analyze T cell polyfunctionality. **e** Single-cell polyfunctionality (left) and polyfunctional strength index (PSI) (right) graphs. Polyfuctionality: co-secretion of 2+ cytokines per cell; PSI: percentage of polyfunctional cells in the sample, multiplied by the intensities of the secreted cytokines. S: spleen. T: tumor. **f** Single-cell polyfunctional heat map illustrates the single-cell cytokine combinations being secreted by each sample. Each column corresponds to a specific cytokine or combination of cytokines, and the orange squares represent the frequency at which the group was secreted by the corresponding sample. The cytokine groups are ordered by overall frequency across all the samples.

To determine the impact of NKTR-214 in combination with ACT on the tumor microenvironment, we performed RNA sequencing (RNA-seq) analysis of tumor samples harvested at day 12. The experiment was conducted two days before the second peak of T-cell expansion in tumors of ACT + NKTR-214 treated mice to assess differences at transcriptomic level potentially responsible for the different cellular phenotype between the two treatments. Hierarchical clustering of the most variable genes demonstrated that ACT + NKTR-214 induced far greater gene expression in tumor samples than ACT + IL-2 treatment (Fig. 5a). The detailed principal component analysis (PCA) showing the clustering of the three biological replicates for all treatment groups is shown in Supplementary Fig 8a. Gene set enrichment analysis (GSEA) of the genes log2 upregulated between ACT + NKTR-214 compared to ACT + IL-2 showed that the top ten significantly enriched sets from Gene Ontology (GO) included immune response, T cell activation and proliferation, and inflammatory-related genes (Fig. 5b). Genes higher expressed in the ACT + NKTR-214 compared to ACT + IL-2 samples include interferon-γ (IFN-γ) (Fig. 5b, Supplementary Fig. 8b), IL-5, IL-6, IL-15, IL-18, and IL-27 (Supplementary Fig. 8b), and chemotaxis-related genes (Fig. 5b, Supplementary Fig. 8c). To better characterize the transcriptional profile of the T cell population, we focused on the expression of selected genes with a known role in regulating T cell populations. As expected, we found an upregulation of key effector genes (*perforin, IFN-γ*, and *granzymes*) in the ACT + NKTR-214 tumors. Increased expression of these genes and *Pdcd1* (PD-1), *Cd28* and *Klrg1* showed the presence of highly cytotoxic effector CD8 T cells in the ACT + NKTR-214 tumors. Over-expression of the inhibitory receptors *Ctla4, Lag3, Havcr2* (Tim-3) and *Tigit*, as well as the transcription factors *Tbx21* (T-bet) and *Eomes*, suggests the increased presence of T cells that had previously signaled through their TCR after NKTR-214 treatment. Interestingly, genes highly expressed in ACT + NKTR-214 tumors also included markers of memory T cells such as *Il7r* (CD127), *Cd44* and *Sell* (CD62L), reflecting the heterogeneity of T cell populations induced by NKTR-214 treatment.

### NKTR-214 increases tumor-specific T cell polyfunctionality

To compare the functional properties of T cells between ACT + NKTR-214 and ACT + IL-2 treated-mice, we evaluated the ability of the adoptively transferred Thy1.1+ CD8 T cells to secrete multiple (>2) cytokines per cell, termed polyfunctionality. We utilized a multiplexed antibody coated chip that allows analyzing thousands of T-cells at the single-cell level for the frequency and intensity of secretion of 28 cytokines (IsoCode Chip, Fig. 5d). Thy1.1+ cells from mice treated in vivo with ACT + NKTR-214 showed an enhanced ability to induce polyfunctional T-cells. In both spleen and tumor, ACT + NKTR-214 increased polyfunctionality by 1.7 and 10 fold, respectively, compared to ACT + IL-2 (Fig. 5e). Though the percentage of cells secreting two cytokines was almost identical in the spleen (2.2% vs. 2.4%, respectively), exposure to NKTR-214 induced a subset of cells to become highly polyfunctional. Zero percent of the adoptively transferred T cells were highly polyfunctional in the spleen samples from mice exposed to ACT + IL-2. In contrast, ACT + NKTR-214 elicited a 21-fold increase in the polyfunctional strength index (PSI, defined as the percentage of polyfunctional cells in the sample, multiplied by the intensities of the secreted cytokines) of Thy 1.1+ CD8 T cells (Fig. 5e). Moreover, ACT + NKTR-214 treatment elicited a 1200-fold increase in PSI of the TILs compared to ACT + IL-2. The increased polyfunctional response by NKTR-214 was driven by the production of the chemoattractive RANTES, followed by MIP-1α, granzyme B, and IFN-γ effector cytokines (Supplementary Fig. 9a). In particular, NKTR-214 increased the secretion frequency and intensity of IFN-γ and MIP-1α in splenic T cells while enhancing granzyme B, MIP-1α, RANTES and decreasing the regulatory/immune suppressive TGF-β in T cells obtained from tumors (Supplementary Fig. 9b, c). The single-cell polyfunctional heatmap shown in Fig. 5f demonstrated the increase in polyfunctional T cell subsets that co-produce combination cytokines in spleen and tumor after NKTR-214.

### Enhanced polyfunctionality of patient’s blood immune cells with NKTR-214 monotherapy

**Fig. 6:**
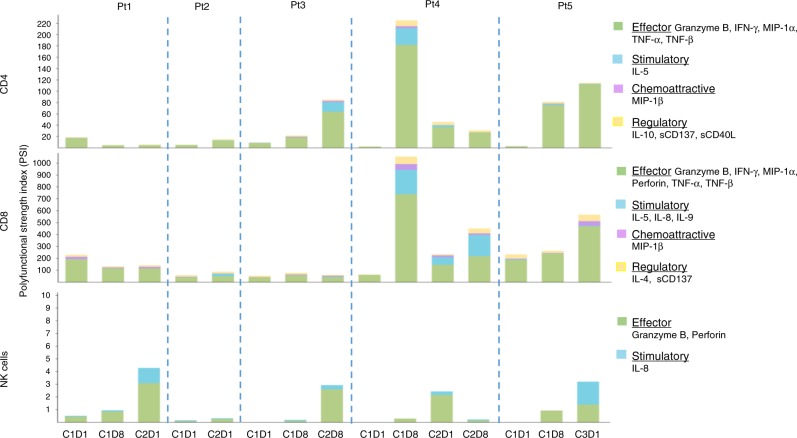
Increase in polyfunctional T and NK cells in PBMC of patients receiving NKTR-214. Single-cell polyfunctional strength index (PSI) computed for CD4, CD8 T cells and NK cells across 5 patients at different time-points. C1D1: cycle 1 day 1; C1D8: cycle 1 day 8; C2D1: cycle 2 day 1; C2D8: cycle 2 day 8; C3D1: cycle 3 day 1.

To explore if the administration of NKTR-214 to humans also induced increased polyfunctional profile of CD4, CD8, and NK cells, as we noted in the mouse model, we used the same single cell polyfunctionality assay chip to analyzed peripheral blood mononuclear cells from five patients treated with NKTR-214 within a phase I dose escalation study (NCT02869295). Results showed that NKTR-214 induced distinct polyfunctional responses in the cell subsets at days eight of cycle one (C1D8), eight of cycle two (C2D1 and C2D8) and one of cycle three (C3D1), compared to pre-dose (cycle one day one, C1D1). NKTR-214 upregulated the PSI of NK cells in blood samples from all the patients after one course of treatment, with a higher increase after the second dose of NKTR-214 (Fig. 6). The PSI of CD4 and CD8 T cells was not affected by the treatment in patient one, while the single-cell polyfunctional strength of CD4 and CD8 T cells in the other four patients was enhanced after NKTR-214 administration, with PSI peaking at various time points in different patients (Fig. 6) demonstrating the importance of serial sampling. Effector and stimulatory cytokines dominated the profiles of all the cell subsets (Fig. 6). Specifically, for CD4 and CD8 T cells the major drivers for increased PSI were TNF-α, IFN-γ, and IL-5 (Supplementary Fig. 10a, b).

## Discussion

In this study, we describe the superior anti-tumor efficacy of NKTR-214 compared to IL-2 to support the antitumor activity of adoptively transferred T cells in a preclinical model. We show that the combined therapy including NKTR-214 was able to control the growth of large, poorly immunogenic tumors and was associated with substantially higher expansion, tumor targeting and persistence of polyfunctional anti-tumor CD8 effector T cells. Increased number of polyfunctional CD8 T cells was also observed in peripheral blood of patients treated with NKTR-214 as a single agent within a phase 1 clinical trial^18^.

Different attempts have been made to use the beneficial effects of the IL-2 pathway while limiting its toxicities to improve therapeutic potential. These approaches include mutated versions of IL-2 with higher binding affinity towards the IL2Rβ^11^, Fc fusion proteins to increase IL-2 serum half-life^19^ and IL-2/anti-IL-2 antibody complexes preferentially activating CD8 effector T cells^20,21^. A recent study set out to improve the efficacy of ACT and mitigate toxicities using engineered orthogonal IL-2 cytokine-orthogonal IL-2-receptor pairs able to bind exclusively with each other, but not with the endogenous counterpart^12^. All these modifications showed promising results in animal models; however, they need to be evaluated in the clinic to assess potential in vivo effects in humans. NKTR-214 is in active clinical testing and its improved pharmacokinetics and pharmacodynamics over IL-2 mitigating immediate over-activation of the immune system and facilitate tolerability^3^. The sequential release of PEG chains avoids the rapid systemic immune activation upon administration while providing sustained signaling preferentially through the IL2Rβγ pathway. The pegylation has the additional ability to decrease the affinity for IL2Rαβγ, constitutive to Tregs, to a greater extent than the affinity for IL2Rβγ, thus favoring the expansion of effector T cells compared to Tregs in the tumor as previously described^3,13^. These combined attributes result in a convenient dosing schedule (every 3 weeks) in human clinical trials. Currently, NKTR-214 is being evaluated clinically in an out-patient setting (NCT02869295) and has shown favorable safety profile^8^.

Interestingly in our studies, the CD8/Tregs ratio was substantially increased in the tumor of mice treated with ACT + NKTR-214, while the ratio was much lower in the spleen, probably due to the homing and preferential expansion of specific pmel-1 effector T cells into the tumor as demonstrated here using several imaging and enumeration methodologies^22^. Local secretion of specific chemokines is thought to favor the migration of T cells into the tumor microenvironment^23^. In our study, transcriptomic profiles showed upregulation of several chemokines and their receptors in tumors of mice treated with ACT + NKTR-214, including CCL1, CCL2, CCL3, CCL4, CCL5, CCL6, CCL7, CCL8, and CXCR3, previously reported to be associated with presence of effector T cells in the tumor site^24^. The adoptively transferred T cells recruited into the tumors of mice treated with NKTR-214 were highly polyfunctional, and capable to simultaneously producing chemoattractive and effector cytokines and to sustain a longer anti-tumor response, as previously reported in clinical trials^25,26^. In addition to enhanced polyfunctionality, these cells delivered high concentrations of cytokines per cell. The ability of the infiltrated T cells to secrete IFN-γ, granzymes, and perforin both in preclinical and clinical setting^18^ supports the ability of NKTR-214 to strongly activate a pool of cytotoxic effector T cells. Interestingly, RNA sequencing data in our mouse model shows the coexistence of T cell effector and exhaustion (or dysfunction) genes with genes related to memory function. This characteristic has been previously described and can be a mechanism to achieve both rapid tumor-cells killing and long-lasting anti-tumor response^27^.

Adoptive T cell transfer therapy with TIL or TCR engineered T cells requires supplemental IL-2 to provide the adequate IL-2-gamma receptor signaling support for brisk T cell proliferation in the host, which is not required with T cells expressing a chimeric antigen receptor (CAR) supposedly due to the provision of costimulatory signaling by the CAR. Together, our results suggest that NKTR-214 supports the expansion and functionality of adoptively transferred T cells. These characteristics, together with low levels of immune suppressive Tregs at the tumor site, are thought to be fundamental for the proper efficacy of immunotherapies^28^. The robust and long-lasting effect of NKTR214 in our preclinical ACT model support its additional potential use in combination with T cell-based adoptively transferred therapeutics.

## Methods

### Mice, cell lines and reagents

C57BL/6 mice (Thy1.2+, The Jackson Laboratory, Bar Harbor, ME) and pmel-1 (Thy1.1+) transgenic mice were generously provided by Dr. Robert Prins, Department of Neurosurgery at the University of California, Los Angeles (UCLA). All mouse model experiments were performed in defined-flora pathogen-free conditions at the AALAC-approved animal facility of the Division of Experimental Radiation Oncology, UCLA following protocol # 2004-159-43I after review and approval by the UCLA Animal Research Committee. B16-F10 is a gp100 positive spontaneous murine melanoma obtained from the American Type Culture Collection (ATCC, Manassas, VA). All the cell lines are mycoplasma-free and were regularly tested for mycoplasma contamination. B16-F10 were authenticated by CellCheck Mouse Test (IDEXX, Westbrook, ME). Cells were cultured with RPMI containing 2 mM L-glutamine (Corning, Corning, NY) with 10% fetal calf serum (Omega Scientific, Tarzana, CA). NKTR-214 was obtained under a material transfer agreement (MTA) with Nektar Therapeutics (San Francisco, CA). Human recombinant IL-2 (aldesleukin) was purchased from Prometheus Therapeutics & Diagnostic (San Diego, CA).

### Pmel-1 ACT in vivo model

C57/BL6 mice were implanted subcutaneously with B16-F10 syngeneic murine melanoma cell line on day-7 and lymphodepleted with 500 cGy of total body irradiation on day-1. Splenocytes from pmel-1 donor mice were depleted of erythrocytes by hypotonic lysis, cultured in RPMI (Corning) culture media with 50 IU/ml mIL-2 (PeproTech, Rocky Hill, NJ) in the presence of 1 μg/ml gp100_25–33_ peptide (AnaSpec, Fremont, CA), and used on days 3–5 after start of the culture. On day 0, when the tumors reached the volume of at least 150 mm^3^, mice received 2 × 10^6^ peptide-activated pmel-1 splenocytes intravenously (i.v.) plus NKTR-214 (0.8 mg/kg, q9dx3, i.v.), IL-2 (0.4 mg/kg, qdX3 every 9 days for 3 cycles, via intraperitoneal injection, i.p.) or vehicle. Tumors were followed by caliper measurements three times per week.

### Immunohistochemistry

Immunohistochemical staining was performed on paraffin-embedded spleen, tumor, kidney and liver samples at the UCLA Anatomic Pathology IHC Laboratory. Sections were cut at 4μm thickness and paraffin removed with xylene and rehydrated through graded ethanol. Endogenous peroxidase activity was blocked with 3% hydrogen peroxide in methanol for 10 min. Heat-induced antigen retrieval (HIER) was carried out for all sections with 0.001 M EDTA buffer, pH = 8.00 using a Biocare decloaker at 95 °C for 25 min. The slides were then incubated for 1 h at room temperature with CD8a (Invitrogen, Carlsbad, CA) at 1/100 dilution, followed by a 30 min incubation with rabbit anti rat secondary. The signal was detected using an anti Rabbit HRP conjugated Polymer (Agilent, Santa Clara, CA) and visualized with the diaminobenzidine reaction. The slides were counterstained with hematoxylin, dehydrated and covered. The imaging and quantification was performed with the HALO Next Generation imaging analysis software (Indica Labs, Corrales, NM). The percentage of CD8 T cells in the slide was automatically counted with the HALO software.

### Bioluminescence imaging

Three days after pmel-1 splenocytes harvest and culture, cells were transduced to express firefly luciferase, using a retroviral vector pMSCV containing a 5′LTR-driven thermostable variant of firefly luciferase, generously provided by Dr. Robert Prins, Department of Neurosurgery at the University of California, Los Angeles (UCLA)^29^. 48 h after transduction, cells were collected, washed and used for ACT. In vivo bioluminescence imaging was performed on tumor-bearing mice for firefly luciferase transduced-Pmel-1 T cell trafficking. Prior to imaging, mice were anesthetized with 2% Isoflurane, injected intraperitoneally with 100 µl of 30 mg/ml of the luciferase substrate, D-Luciferin (Xenogen Corp., Alameda, CA) in PBS. Mice were shaved to minimize the amount of light absorbed by black fur. The Xenogen IVIS 200 Imaging System (Xenogen/Caliper Life Sciences, Waltham, MA) was used to detect photon emission from tumor-bearing mice with an acquisition time of 0.5–1 min^17^. Analyses of the images were performed using Living Image software (Xenogen) and Igor Image analysis software (Wave Metrics, Lake Oswego, OR) by drawing regions of interest over the region and obtaining maximum values in photons/second/cm^2^/steradian.

### Immuno-PET imaging and ex vivo biodistribution

Non-invasive immuno-PET tracking CD8 T cells in vivo was performed using ^89^Zr-desferrioxamine-labeled anti-CD8 cys-diabody (cDb) at the UCLA Preclinical Imaging Technology Center, Crump Institute^30^. For microPET imaging, 150 µL doses containing Zr-89 radiolabeled cDb were prepared in saline for i.v. injection. Mice were anesthetized using 2% isoflurane and microPET scans were acquired using an Inveon microPET scanner (Siemens, Munich, Germany) followed by microCT scan (ImTek, Wedesboro, NJ). MicroPET images were reconstructed using non-attenuation or scatter corrected maximum a posteriori (MAP) reconstruction and AMIDE was used for image analysis and display^31^. At 24 h post-injection, mice were euthanized, the organs and blood were collected, weighed and the radioactivity was counted to assess CD8 T cells biodistribution. The percent-injected dose per gram of tissue (%ID/g) was calculated using a standard containing one percent of the injected dose^31^.

### Mass cytometry

Tumors harvested from mice treated with ACT + IL-2 or ACT + NKTR-214 were digested using the Tumor Dissociation Kit, mouse (Miltenyi, Bergisch Gladbach, Germany). Spleen were dissociated through a 70 μm cell strainer and red blood cells lysed using the ACK lysis buffer (Lonza, Basel, Switzerland). Splenocytes and tumor cells staining and data acquisition were performed using a protocol adapted from Wei and colleagues^32^. Metal-conjugated antibodies were purchased from Fluidigm (San Francisco, CA), Biolegend and Thermo Fisher or conjugated to unlabeled antibodies at the UCLA Flow Cytometry Core. The mass cytometry staining panel is described in Supplementary Table 1. Cells were washed in PBS and resuspended at a concentration of 1–5 × 10^6^ cells/mL in serum-free RPMI (Corning) and live–dead stained with 5 μmol/L cisplatin (Fluidigm) for 5 min at 37 °C. Cells were washed and stained with a surface protein antibody cocktail for 30 min at room temperature, and were then washed and spun for 10 min at 4 °C. Cells were then fixed using a FoxP3/transcription factor–specific fixation buffer (eBioscience, Santa Clara, California) for 1 h at room temperature, followed by addition of the FoxP3/transcription factor permeabilization buffer, and were spun for 10 min at 4 °C. Cells were then stained with an intracellular antibody cocktail for 1 h at room temperature. Finally, cells were incubated for 1 h at room temperature with 250 nmol/L iridium intercalator (Fluidigm) to label cellular DNA. Cells were then washed with PBS, and finally with distilled water. Samples were interrogated using a Helios mass cytometer (Fluidigm, San Francisco, CA) at the UCLA Flow Cytometry Core Facility. Samples were manually gated in FlowJo by stability of time, cells with no beads (Ir193^+^/Ce140^−^), cleanup (double positive for DNA), singlets (Ir193^+^), live (195Pt^−^/CD45^+^) and by the desired expression markers (CD45 + or (CD45 + Cd11b- Cd11c- CD19- CD161- to gate the T cells) for each particular analysis (Supplementary Fig. 6a). Mass cytometry data analysis was performed using the open-source R/Bioconductor package cytofkit^33^. Individual sample data were subsampled to 5,000 events. We set this cut-off based on the reasoning that 5000 events would be sufficient for phenotypic clustering and dimension reduction analyses. PhenoGraph clustering and t-Distributed Stochastic Neighbor Embedding (t-SNE) plots were performed using cytofkit for cell subsets detection, visualization, and interpretation. For t-SNE plots overlaid with expression of individual parameters, signal intensity is displayed in arcsinh transformed values.

### RNA extraction, sequencing, and analysis

Total RNA extraction was performed using the RNeasy Mini Kit (Qiagen, Hilden, Germany) from B16-F10 tumors treated with ACT + IL-2 and ACT + NKTR-214. RNA sequencing was performed using the Illumina HiSeq 3000 platform on 50-bp single-end libraries at the UCLA technology Center for Genomics & Bioinformatics. Reads were mapped using HISAT2 version 2.0.4^34^ and aligned to the Mus musculus genome NCBI build GRCm38. Reads were quantified HTSeq version 0.6.1^35^ and normalized to fragments per kilobase of exon per million fragments mapped (FPKM) expression values. FPKM values were then log2 transformed with an offset of 1. Hierarchical clustering was performed on z-scored gene expression values with complete linkage using the Euclidean distance metric using R (http://www.R-project.org/). To identify pathways enriched in the ACT + NKTR-214-treated tumors, Gene Set Enrichment Analysis (GSEA) was performed using the pre-ranked option. Genes were ranked by log2 fold changes between ACT + IL-2 and ACT + NKTR-214. Enrichment was assessed across the curated Molecular Signatures Database C5 GO biological process gene sets^36^.

### Single-cell multiplex cytokine profiling of murine T cells

CD8 pmel-1 (Thy-1.1+) T cells from spleen and tumors of mice treated with ACT + IL-2 or ACT + NKTR-214 (3 mice/group) were collected at day 7 after treatment, pooled and sorted using Alexa Fluor 700 anti-mouse CD90.1 (Thy1.1), clone OX-7 antibody (Biolegend, San Diego, CA). Sorted cells were stimulated with immobilized anti-mouse CD3 (Invitrogen) and soluble anti-mouse CD28 (Invitrogen) at 37 °C, 5% CO_2_ for 48 h. After stimulation, cells were stained with Alexa Fluor 647-conjugated anti-mouse CD8 (Biolegend) at room temperature for 20 min. Approximately 30,000 T cells were loaded onto an IsoCode chip (IsoPlexis, New Heaven, CN) containing ~12,000 microchambers pre-patterned with a 28-plex antibody array, imaged for single cell location in microchambers and incubated at 37 °C, 5% CO_2_ for additional 16 h. Following incubation period, ELISA detection was used to determine which combinations of proteins were being secreted by each individual cell. Secreted proteins from single cells were captured by antibody-barcoded slides; the polyfunctional profile (2+ proteins per cell) of single cells was evaluated by IsoPlexis’ software.

### Patient samples

Fifteen cryopreserved peripheral blood mononuclear cells (PBMC) from patients with histologically confirmed locally advanced or metastatic solid tumors were provided by the Department of Melanoma Medical Oncology, Division of Cancer Medicine, University of Texas MD Anderson Cancer Center, Houston, TX. This study is a phase I dose escalation study, identified as NCT02869295 on www.clinicaltrial.gov. We are not reporting the results of the clinical trial, in this article, we report the analyses on blood cell samples obtained from a subset of five subjects. Patients were treated with 0.003 or 0.006 mg per kg body weight (mg/kg) every 2 weeks or every 3 weeks as a 15 –min i.v. infusion. Blood to obtain PBMC was collected on the first day of treatment (C1D1), at week 1 after treatment (C1D8), at day one and eight of cycle two (C2D1 and C2D8) and at day one of cycle three (C3D1). The study was approved by the institutional review board of University of Texas MD Anderson Cancer Center. Informed consent was obtained from all participants.

### Single-cell multiplex cytokine profiling of human PBMC

PBMCs from five patients were thawed and recovered in the complete RPMI media (Thermo Fisher Scientific, Waltham, MA) with 10 ng/ml IL-2 (Biolegend) at 37 °C, 5% CO_2_. After overnight incubation, viable cells were enriched by Ficoll. CD4+ T cells, CD8 T cells and NK cells were separated by anti-CD4, anti-CD8 or anti-CD56 microbeads (Miltenyi). Enriched NK cells were labeled with Carboxyfluorescein succinimidyl ester (CFSE, Thermo Fisher Scientific), rinsed and resuspended in RPMI media at a density of 1 × 10^6^/ml with addition of PMA (5 ng/ml, Sigma-Aldrich) and Ionomycin (500 ng/ml, Sigma-Aldrich) to be loaded onto an IsoCode chip (IsoPlexis). Enriched CD4 and CD8 T cells were resuspended in fresh complete RPMI media at 1 × 10^6^/ml and activated with immobilized anti-human CD3 (10 ug/ml, Thermo Fisher Scientific) and soluble anti-human CD28 (5 ug/ml, Thermo Fisher Scientific) in a 96-well flat-bottom plate (Corning Life Science) at 37 °C, 5% CO_2_ for 24 h. After stimulation, cells were stained with PE-conjugated anti-human CD4 (Biolegend) or Alexa Fluor 647-conjugated anti-human CD8 (Biolegend) at room temperature for 20 min and loaded onto an IsoCode chip. Each IsoCode chip contains ~12,000 microchambers pre-patterned with a full copy of 32-plex antibody array including Effector: Granzyme B, TNFα, IFN-γ, MIP1α, Perforin, TNFβ; Stimulatory: GM-CSF, IL-2, IL-5, IL-7, IL-8, IL-9, IL-12, IL-15, IL-21; Chemoattractive: CCL11, IP-10, MIP-1β, RANTES; Regulatory: IL-4, IL-10, IL-13, IL-22, sCD137, sCD40L, TGFβ1; Inflammatory: IL-6, IL-17A, IL-17F, MCP-1, MCP-4, IL-1β. The polyfunctional profile (2+  proteins per cell) of single cells was evaluated by IsoSpeak software.

### Statistical analyses

Data are presented as mean ± SEM and representative of at least two independent experiments. In tumor growth studies the significance between experimental groups (ACT + vehicle, ACT + IL-2 and ACT + NKTR-214) was defined using ANOVA, with the Bonferroni-Dunn multiple comparison post-test. Differences in Kaplan–Meier survival curves showing tumor delay (time to achieve tumor volume of 1000 mm^3^) were calculated using Mantel–Cox test. *P*-values were calculated using two-way ANOVA (Tukey’s multiple comparison) to compare the intensity of ROI in BLI experiments. Differences between NKTR-214 and IL-2 were analyzed using unpaired Student’s *t*-test. *P*-values < 0.05 were considered significant. Statistical analyses were performed using GraphPad Prism version 7.03 (GraphPad Software).

### Reporting summary

Further information on research design is available in the Nature Research Reporting Summary linked to this article.

## Supplementary information

Supplementary Information

Reporting Summary

## Data Availability

The authors state that all data generated during this study are within the article and its supplementary information files and from the corresponding author upon reasonable request. The RNA sequencing data have been deposited in the GEO database under the accession code GSE118748. A reporting summary for this article is available as a Supplementary Information file.

## Source data

Source Data
