# Supplementary material for: Persistence of adoptively transferred T cells with a kinetically engineered IL-2 receptor agonist
Source: Nat Commun. 2020 Jan 31;11:660. doi: 10.1038/s41467-019-12901-3 (PMC6994533; doi:10.1038/s41467-019-12901-3)
Supplement: Supplementary file 1 — Supplementary Information [file 41467_2019_12901_MOESM1_ESM.pdf]

Supplementary Information

Persistence of Adoptively Transferred T cells with a Kinetically Engineered IL-2 Receptor Agonist

Parisi G. et al.,

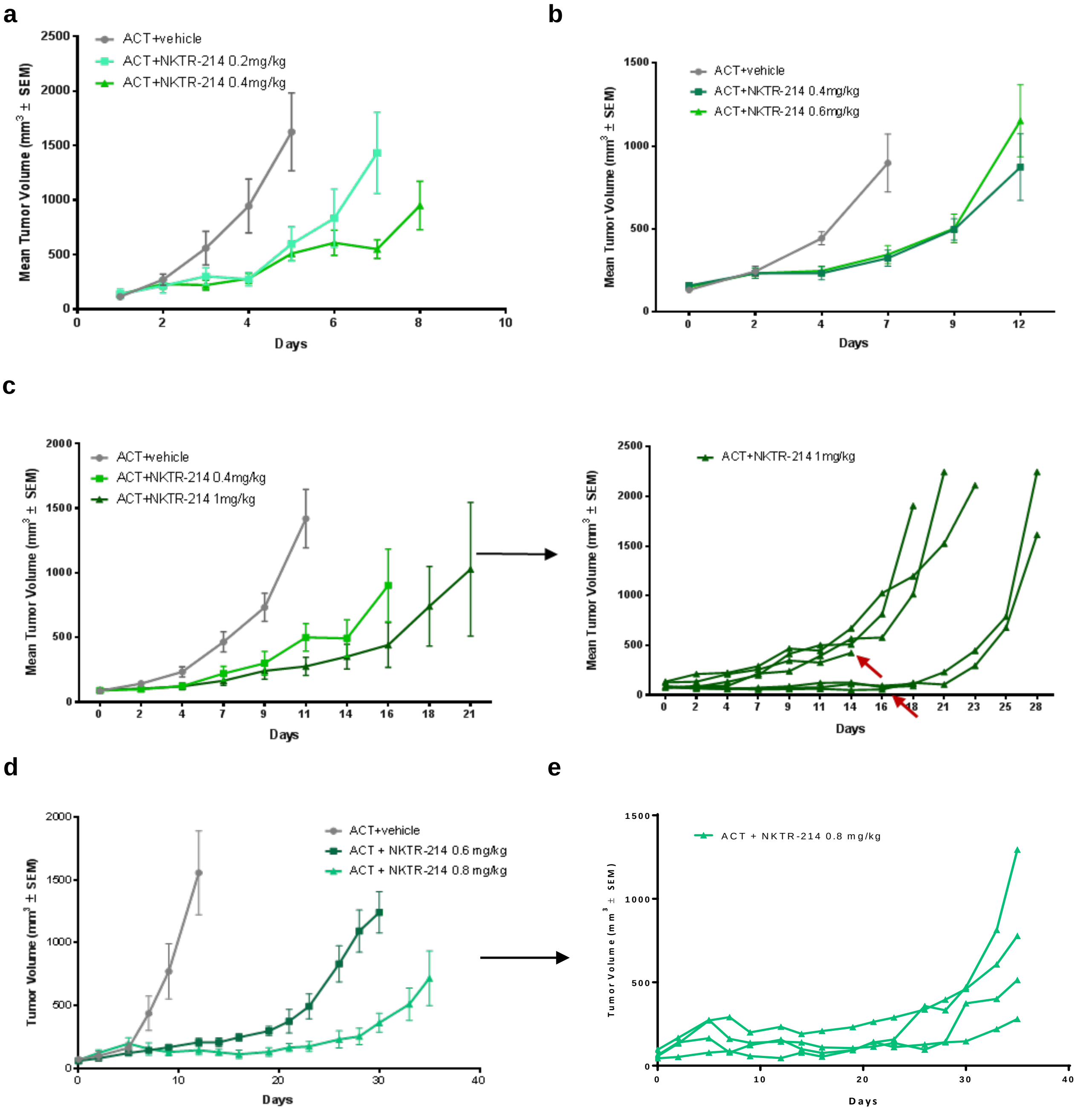

Figure S1. Dose escalation of NKTR-214 in combination with ACT to evaluate tumor antitumor activity and toxicity. (**a**, **b, c, d**) Mice treated with 0.2, 0.4, 0.6, 0.8 and 1 mg/kg given once every 9 days for three doses (q9dx3) in four consecutive experiments. All mice survived and did not show signs of toxicities in (**a**), (**b**) and (**d**), n=4 in **a** and **d**, n=8 in **b**. (**c**) Two mice died in the group treated with 1 mg/kg, right graph, pointed by the red arrows, leading to the definition of 0.8 mg/kg as the maximum tolerated dose, n=7. (**e**) The dose of 0.8 mg/kg was administered either once every 9 days for three doses (q9dx3) or every 9 days for four doses (q9dx4). No difference was shown in the 2 dose schedules, n=4.

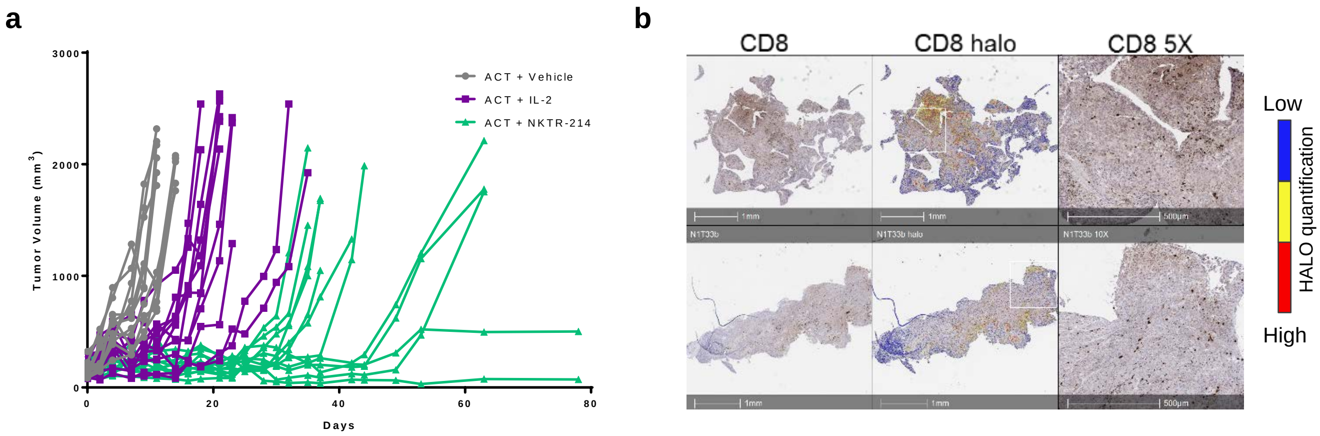

Supplementary Figure 2. NKTR-214 in combination with ACT induced stable disease. **a** Changes in tumor volume of single mice over time. **b** CD8+ IHC staining of FFPE tumor of two mice showing stable disease (collected at day 78 after ACT). HALO software analysis showing intensity of marker expression, from low (blue) to high (red) expression level.

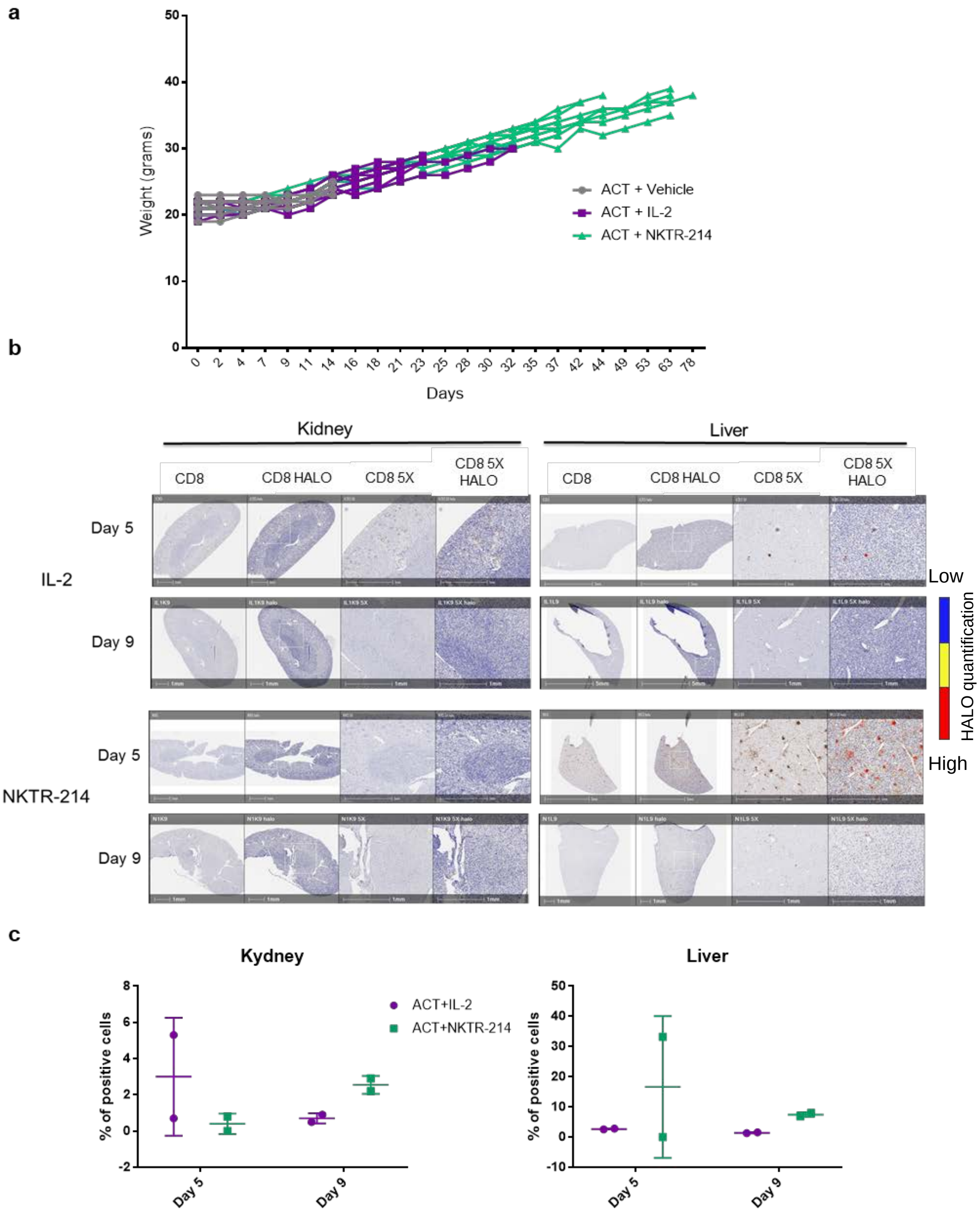

Supplementary Figure 3. No evidence of significant toxicity in mice. **a** Weight of single animals treated with ACT+vehicle, IL-2 or NKTR-214. **b** CD8+ IHC staining of FFPE kidney and liver at different time points. HALO software analysis showing intensity of marker expression, from low (blue) to high (red) expression level. **c** HALO software quantification of IHC slides showing the percentage of CD8 T cell expansion in kidney and liver at different time points. Mean ± s.e.m.

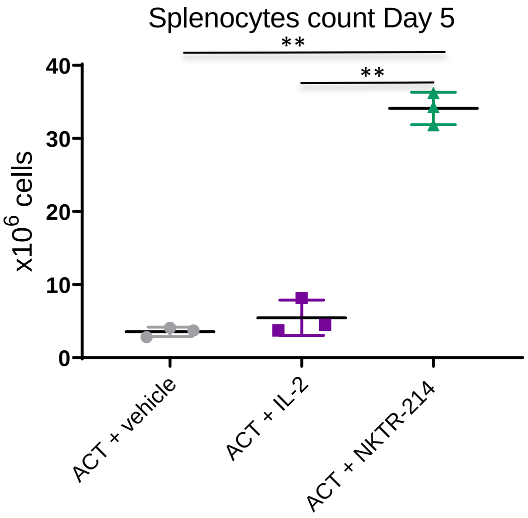

Supplementary Figure 4. Splenocytes counts at day 5 after treatment. NKTR-214 induced a rapid splenic cell repopulation after irradiation. Mean ± s.e.m (n=3), unpaired t test (** p<0.0001 with bars indicating comparisons).

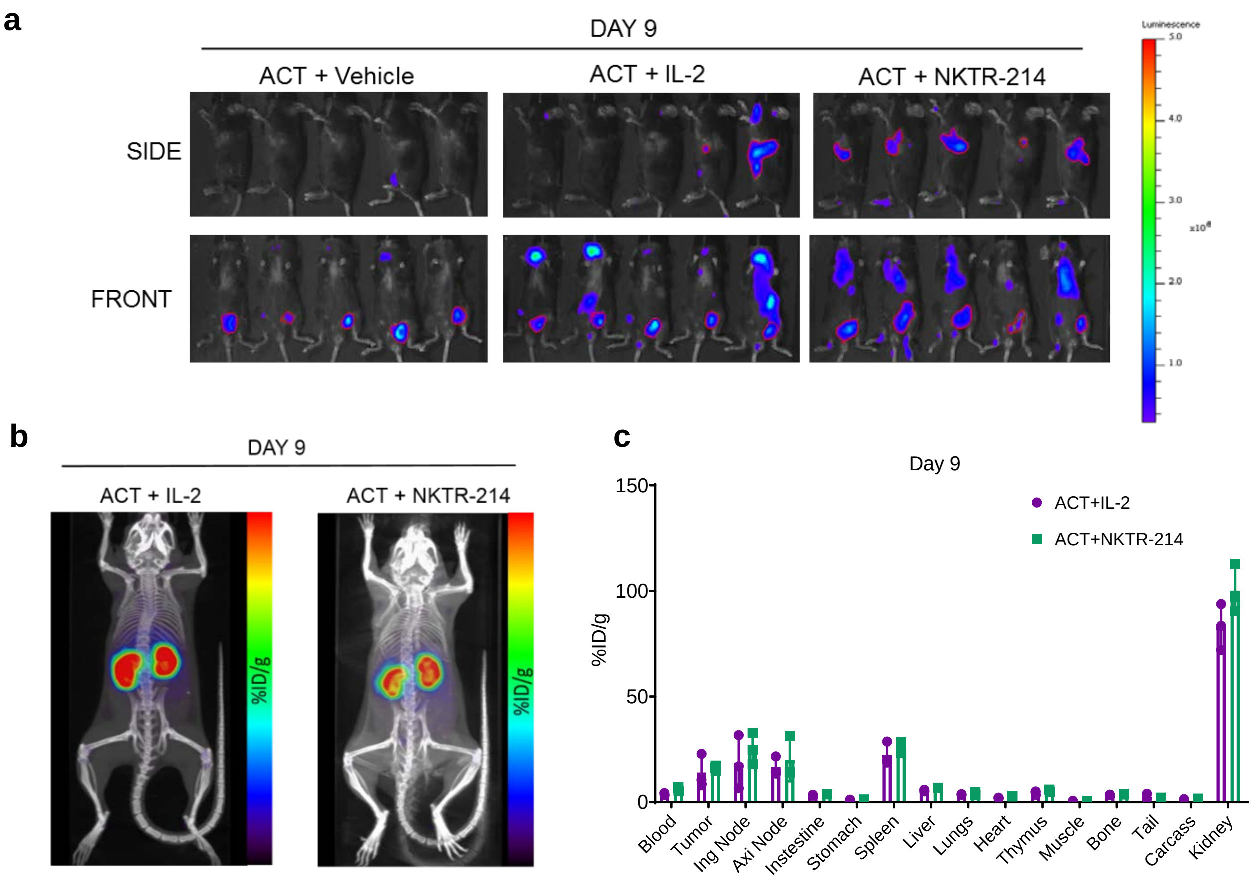

Supplementary Figure 5. Decreased CD8 T cells expansion at day 9 after treatment. **a** Time-course of bioluminescence imaging (BLI) of T cell trafficking *in vivo*. Representative figures on days 9, five replicate mice per group. T cell expansion in spleen (upper panels, SIDE); mobilization and persistence in tumor (lower panels, FRONT).). Scale bar shows the radiance, going from a minimum of $3.0x10^5$ e to a maximum of $5.0x10^6$ photons $second^{-1}$ cm^{-2} $steradian^{-1}$. **b** Representative immuno-PET/CT images acquired on day 9 after treatment with ACT+IL-2 or ACT+NKTR-214 (n=3/group). I.v. injection of Zr-89 labeled anti-CD8 cys-diabodies (cDb) was performed 24 hours prior to imaging the C57/BL6 mice. Scale bar represents the percent-injected dose per gram of tissue (%ID/g) detected, from low (black) to high (red) intensity. **c** Biodistribution at 24 hours post-injection of Zr-89 cDb. %ID/g: injected dose per gram. The diabody and residualizing radionucleotide undergo renal clearance, hence signals from the kidney are not considered in the analysis. Mean ± s.e.m (n=3).

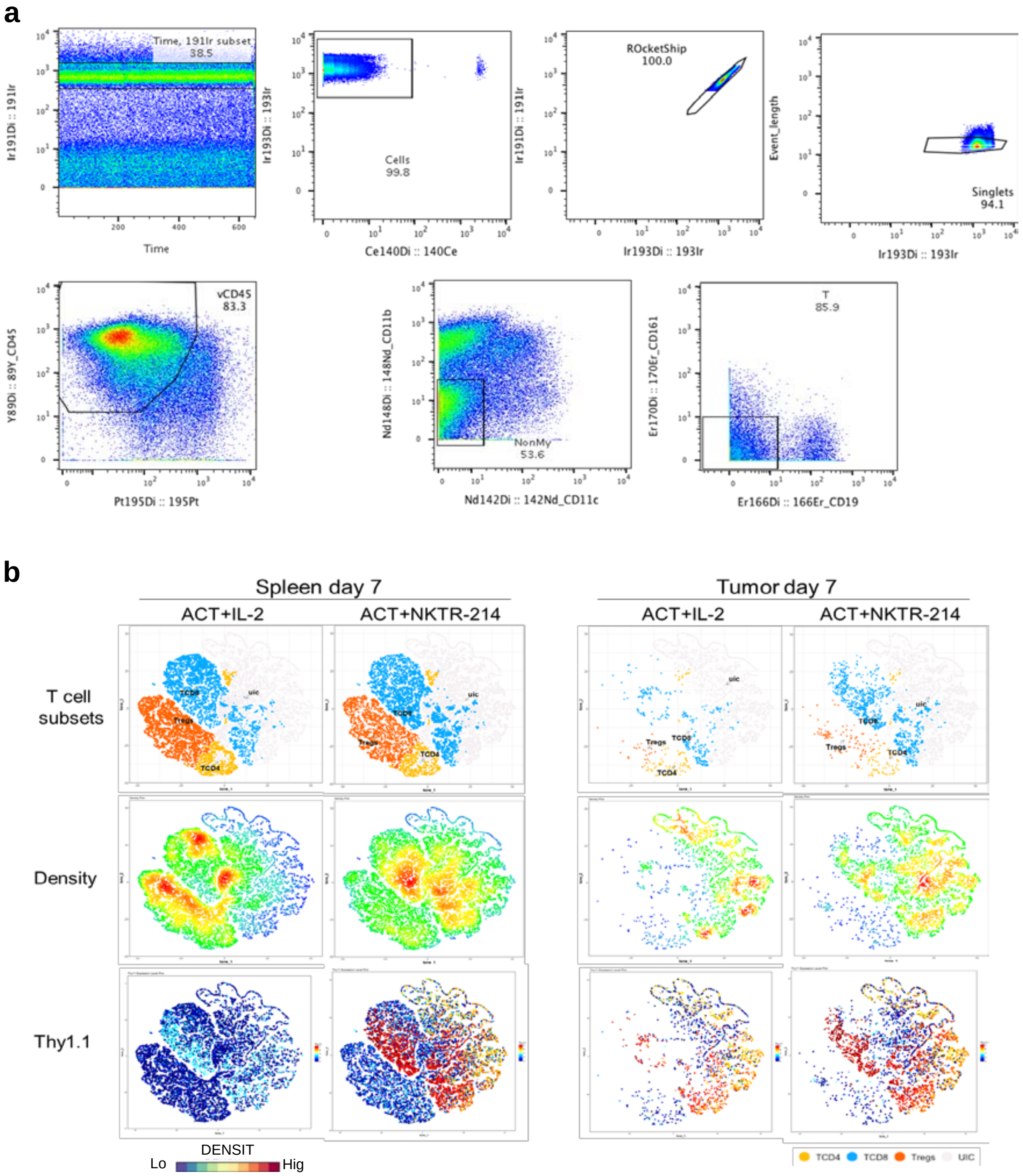

Supplementary Figure 6. Manual gating strategy for mass cytometry analysis. **a** Samples were manually gated in FlowJo by event Samples were manually gated in FlowJo by stability of time, cells with no beads (Ir193^{+}/ Ce140^{-}), cleanup (double positive for DNA), singlets (Ir193^{+}), live (195Pt^{-}/CD45^{+}) and by the desired expression markers (CD45+ or (CD45+ Cd11b- Cd11c- CD19- CD161- to gate the T cells) for each particular analysis. **b-c** T-SNE plots showing annotated clusters (upper panels), density plots (middle panels) and Thy1.1 marker expression level plot (lower panels) in spleen (**b**) and tumor (**c**) at day 7. Density scale bar represents marker expression of cells for a given cluster, ranging from low expression (blue) to high expression (red).

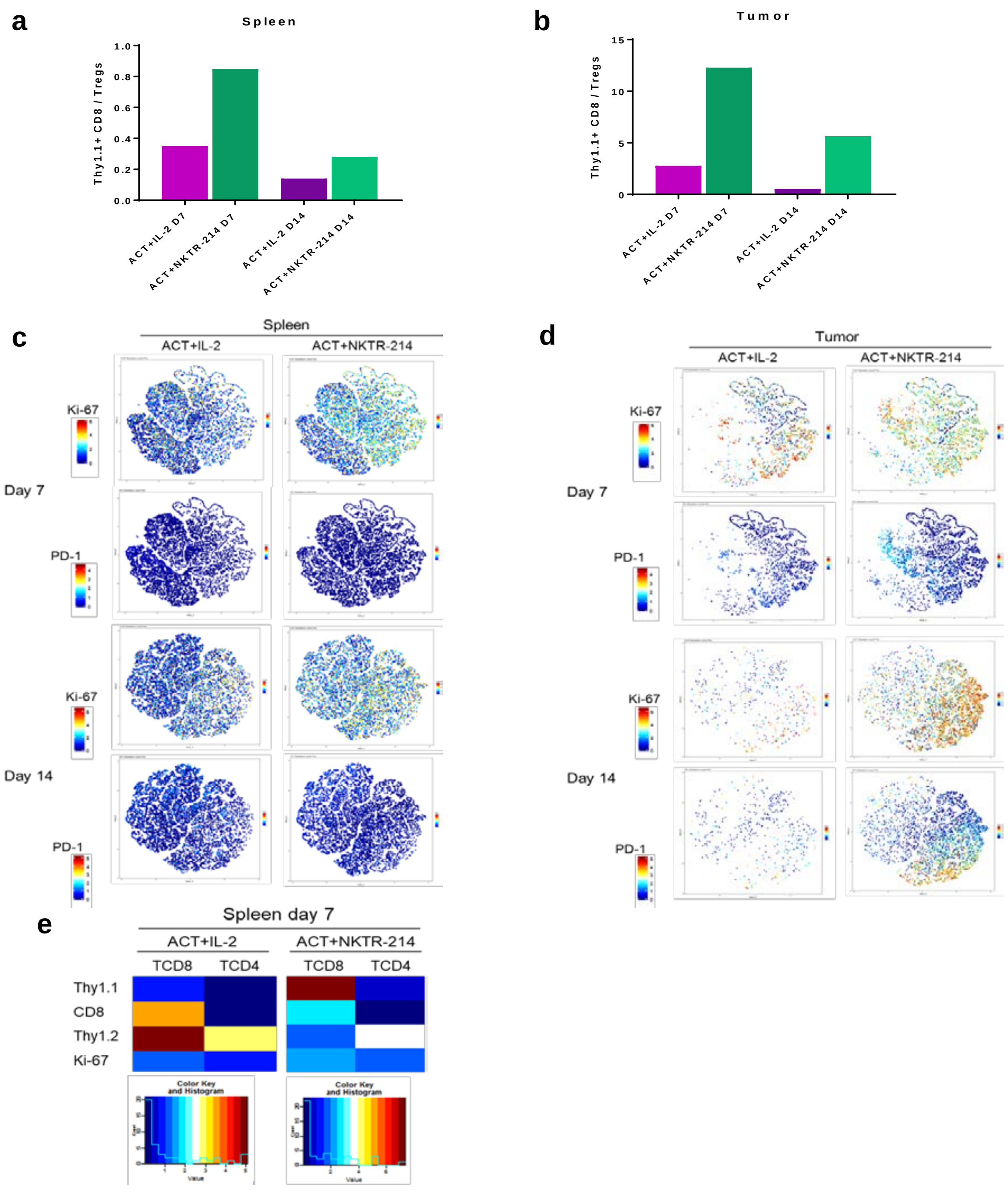

Supplementary Figure 7. NKTR-214 increased the CD8 T vs Tregs ratio. Ratio of Thy1.1, TCD8 and Tregs in spleen **a** and tumor **b**. **c-d** T-SNE plots showing K i-67 (upper panels) and PD-1 marker expression level plot (lower panels) in spleen (**c**) and tumor (**d)** at day 7 and 14. Density scale bar represents marker expression of cells for a given cluster, ranging from low expression (blue) to high expression (red). **e** Heatmap displaying median marker expression of CD4 and CD8 in spleen of ACT+IL-2 or ACT+NKTR-214 treated mice at day 7.

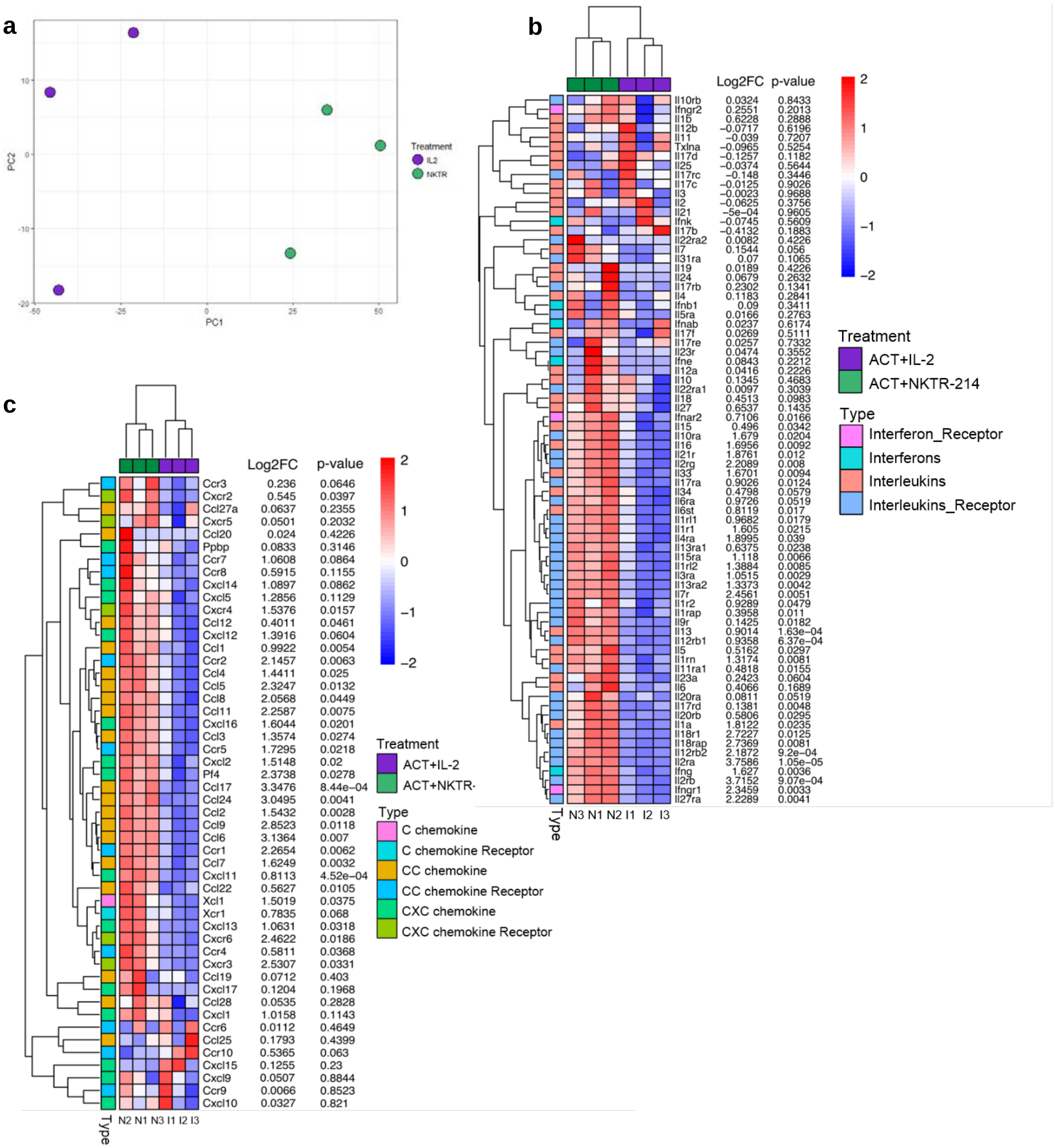

Supplementary Figure 8. RNA-Seq analysis of tumors. **a** PCA of gene expression profile of the tested samples. **b-c** Gene expression heatmap of interferons, interleukins and their receptors (**b**) and chemokines and their receptors (**c**).

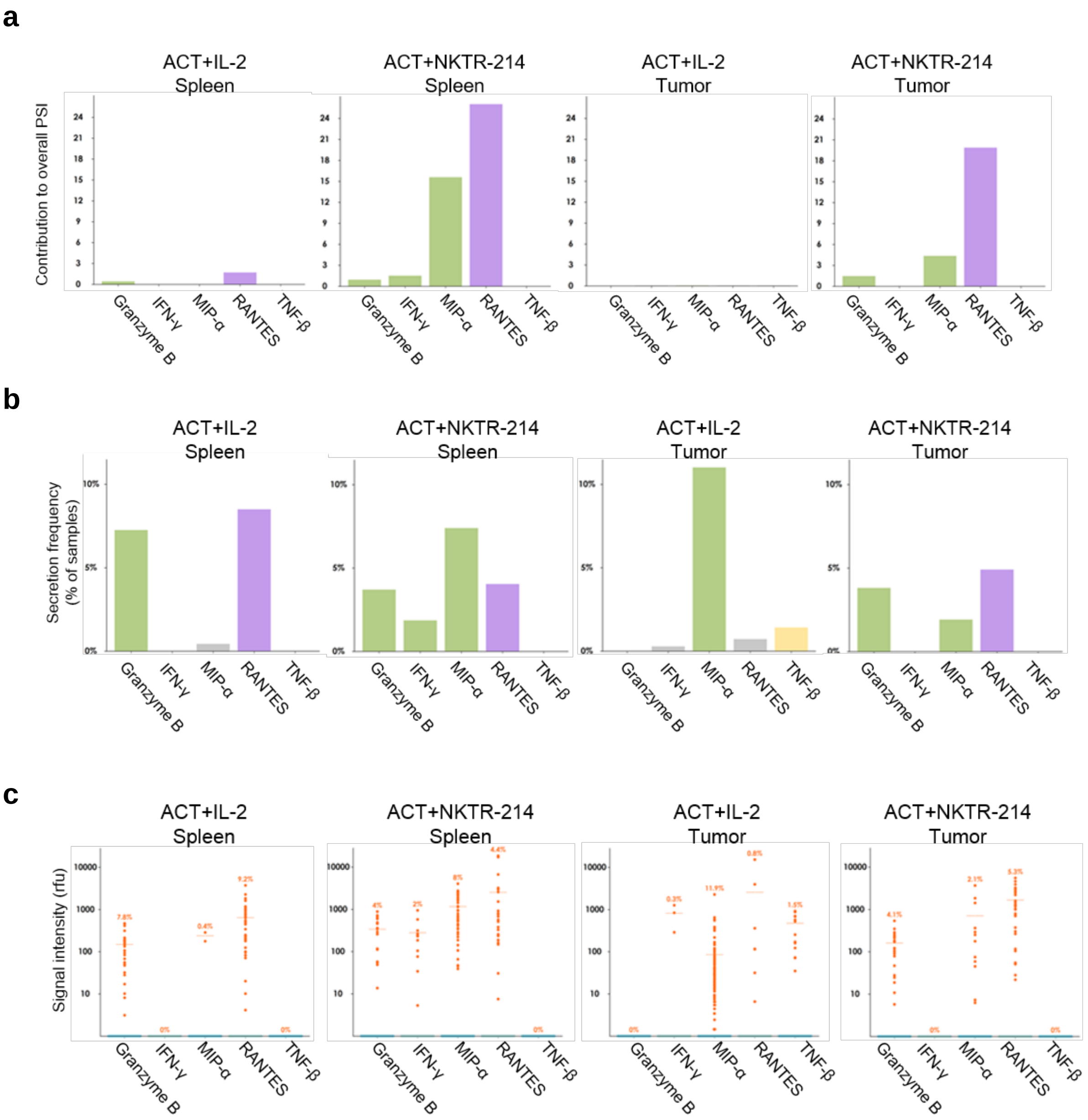

Supplementary Figure 9. Polyfunctionality assay analysis of adoptively transfer Thy1.1. **a** Single-cell polyfunctional strength composition showing which individual cytokines are driving the response. **b** Cytokine secretion graphs revealing the frequency at which each cytokine was secreted by single cells in each sample (without factoring in the secretion intensities). **c** Signal distribution graphs showing the single-cell secretion intensity of each cytokine in each sample.

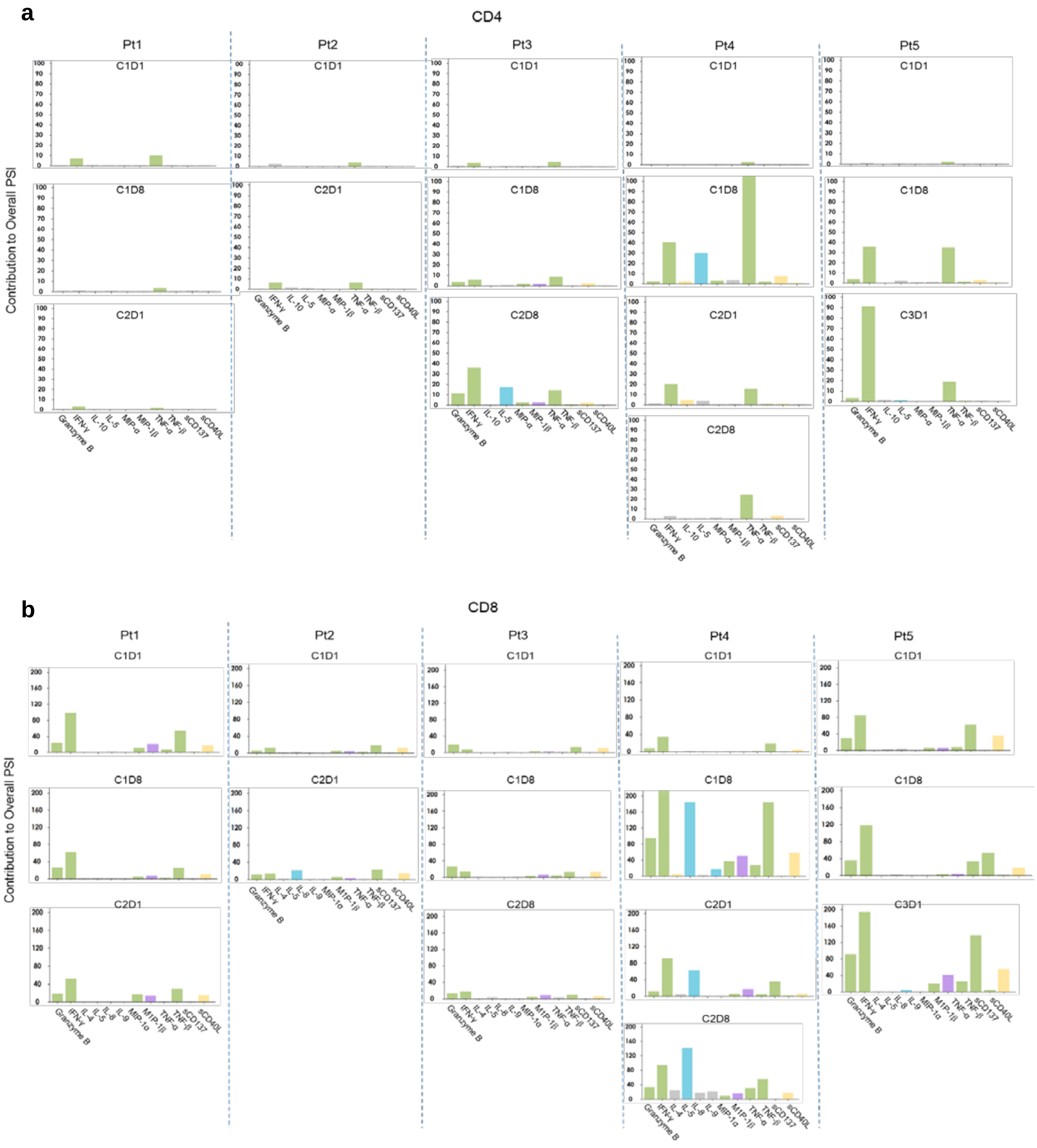

Supplementary Figure 10. Single-cell polyfunctional strength (PSI) composition. **a-b** Single-cell polyfunctional strength composition showing which individual cytokines are driving the response in CD4 (**a**) and CD8 (**b**) T cells.

Supplementary Table 1. Staining panel for Mass Cytometry

Label	Target	Clone	Dilution	Source	CAT #
		**Surface**			
89Y	CD45	30-F11	1/100	Fluidigm	3089005B
142Nd	CD11c	N418	1/100	Fluidigm	3142003B
143 Nd	CD69	H1.2F3	1/100	Fluidigm	3143004B
148Nd	CD11b	M1/70	1/100	Fluidigm	3148003B
149Sm	CD62L (L-selectin)	MEL-14	1/100	Biolegend	104443
152Sm	CD3e	145-2C11	1/100	Fluidigm	3152004B
155Gd	CD25	3C7	1/100	Biolegend	101913
156Gd	CD90.2 (Thy-1.2)	30-H12	1/100	Fluidigm	3156006B
159Tb	CD279_PD1	29F.1A12	1/100	Fluidigm	3159024B
162Dy	CD90.1(Thy1.1)	OX-7	1/100	Biolegend	202501
166Er	CD19	**6D5**	1/100	Fluidigm	3166015B
168Er	CD8	53-6.7	1/100	Fluidigm	3168003B
170Er	CD161 (NK1.1)	PK136	1/100	Fluidigm	3170002B
171Yb	CD44	IM7	1/100	Fluidigm	3171003B
172Yb	CD4	RM4-5	1/100	Fluidigm	3172003B
174Yb	MCH_II_IA_IE	M5/114.15.2	1/100	Fluidigm	3174003B
		**Intracellular**			
115 IN	Ki67	SolA15	1/100	eBiosciences	14-5698-82
141Pr	TNF alfa	MP6-XT22	1/100	Fluidigm	3141013B
147Sm	Eomes	Dan11mag	1/100	Thermo	14-4875-82
158Gd	FoxP3	FJK-16s	1/100	Fluidigm	3158003A
161Dy	t-bet	4B10	1/100	Fluidigm	3161014B
165Ho	IFNgamma	XMG1.2	1/100	Fluidigm	3165003B
