# Supplementary material for: Persistence of adoptively transferred T cells with a kinetically engineered IL-2 receptor agonist
Source: Nat Commun. 2020 Jan 31;11:660. doi: 10.1038/s41467-019-12901-3 (PMC6994533; doi:10.1038/s41467-019-12901-3)
Supplement: Supplementary file 2 — Reporting Summary [file 41467_2019_12901_MOESM2_ESM.pdf]

nature research

Corresponding author(s): Antoni Ribas

Last updated by author(s): 08/20/2019

Reporting Summary

Nature Research wishes to improve the reproducibility of the work that we publish. This form provides structure for consistency and transparency in reporting. For further information on Nature Research policies, see Authors & Referees and the Editorial Policy Checklist .

Statistics

For all statistical analyses, confirm that the following items are present in the figure legend, table legend, main text, or Methods section.

n/a	Confirmed	
☐	☒	The exact sample size (*n*) for each experimental group/condition, given as a discrete number and unit of measurement
☐	☒	A statement on whether measurements were taken from distinct samples or whether the same sample was measured repeatedly
☐	☒	The statistical test(s) used AND whether they are one- or two-sided<br>*Only common tests should be described solely by name; describe more complex techniques in the Methods section.*
☒	☐	A description of all covariates tested
☐	☒	A description of any assumptions or corrections, such as tests of normality and adjustment for multiple comparisons
☐	☒	A full description of the statistical parameters including central tendency (e.g. means) or other basic estimates (e.g. regression coefficient) AND variation (e.g. standard deviation) or associated estimates of uncertainty (e.g. confidence intervals)
☒	☐	For null hypothesis testing, the test statistic (e.g. *F*, *t*, *r*) with confidence intervals, effect sizes, degrees of freedom and *P* value noted<br>*Give P values as exact values whenever suitable.*
☒	☐	For Bayesian analysis, information on the choice of priors and Markov chain Monte Carlo settings
☐	☒	For hierarchical and complex designs, identification of the appropriate level for tests and full reporting of outcomes
☒	☐	Estimates of effect sizes (e.g. Cohen's *d*, Pearson's *r*), indicating how they were calculated

Our web collection on statistics for biologists contains articles on many of the points above.

Software and code

Policy information about availability of computer code

Data collection	No software was used for Data collection
Data analysis	The imaging and quantification was performed with the HALO Next Generation imaging analysis software (Indica Labs).<br>Bioluminescence imaging was carried out with a Xenogen IVIS 200 Imaging System (Xenogen/Caliper Life Sciences).<br>Micro-PET scans were acquired using an Inveon microPET scanner (Siemens), followed by micro-CT scans (ImTek). MicroPET images were reconstructed using non attenuation or scatter corrected filtered back-projection, and AMIDE was used for image analysis and display.<br>Mass cytometry data analysis was performed using the open-source R/Bioconductor package cytofkit.<br>Reads were mapped using HISAT2 version 2.0.431 and aligned to the Mus musculus genome NCBI build GRCm38. Reads were quantified HTSeq version 0.6.1.<br>The polyfunctional profile (2+ cytokines secreted per cell) of single cells was evaluated by IsoSpeak software.<br>Statistical analyses were performed using GraphPad Prism version 7.03 (GraphPad Software).

For manuscripts utilizing custom algorithms or software that are central to the research but not yet described in published literature, software must be made available to editors/reviewers. We strongly encourage code deposition in a community repository (e.g. GitHub). See the Nature Research guidelines for submitting code & software for further information.

Data

Policy information about availability of data

All manuscripts must include a data availability statement. This statement should provide the following information, where applicable:

- Accession codes, unique identifiers, or web links for publicly available datasets
- A list of figures that have associated raw data
- A description of any restrictions on data availability

RNA sequencing data has been deposited in GEO repository under the accession number GSE118748.

Field-specific reporting

Please select the one below that is the best fit for your research. If you are not sure, read the appropriate sections before making your selection.

☒ Life sciences ☐ Behavioural & social sciences ☐ Ecological, evolutionary & environmental sciences

For a reference copy of the document with all sections, see nature.com/documents/nr-reporting-summary-flat.pdf

Life sciences study design

All studies must disclose on these points even when the disclosure is negative.

Sample size	No statistical methods were used to determine sample size. The numbers of animals per group are based on the need for proper statistical evaluation, which is based on our experience and the experience of other investigators.
Data exclusions	No data were excluded.
Replication	All attempts at replication were successful. Number of replicates per experiment are described in each figure legend.
Randomization	The mice were randomly assigned in the different groups.
Blinding	Investigators were not blinded to group allocation for the majority of experiments performed, except for tumor measurements. All forms of data collected, such as body weight, survival, BLI signal, cytokine secretion, etc could be measured objectively.

Reporting for specific materials, systems and methods

We require information from authors about some types of materials, experimental systems and methods used in many studies. Here, indicate whether each material, system or method listed is relevant to your study. If you are not sure if a list item applies to your research, read the appropriate section before selecting a response.

Materials & experimental systems

n/a	Involved in the study	
☐	☒	Antibodies
☐	☒	Eukaryotic cell lines
☒	☐	Palaeontology
☐	☒	Animals and other organisms
☒	☐	Human research participants
☐	☒	Clinical data

Methods

n/a	Involved in the study	
☒	☐	ChIP-seq
☒	☐	Flow cytometry
☒	☐	MRI-based neuroimaging

Antibodies

Antibodies used	Supplementary table 1 provides all the information needed.
Validation	Validation of the antibodies was performed by UCLA Jonsson Comprehensive Cancer Center (JCCC) and Center for AIDS Research Flow Cytometry Core Facility

Eukaryotic cell lines

Policy information about cell lines

Cell line source(s)	B16-F10 from the American Type Culture Collection (ATCC).

Authentication	Cell lines were authenticated by IDEXX using CellCheck Mouse method, combining STR-based DNA profiling and multiplex PCR to detect both contamination and misidentification.
Mycoplasma contamination	Cell lines tested negative for mycoplasma contamination.
Commonly misidentified lines (See ICLAC register)	No commonly misidentified cell lines were used.

Animals and other organisms

Policy information about studies involving animals; ARRIVE guidelines recommended for reporting animal research

Laboratory animals	C57BL/6J (B6) mice were 6-8 week old females. Pmel-1 (Thy1.1+) transgenic mice were 5-7 week old females provided by Dr. Robert Prins, Department of Neurosurgery at the University of California, Los Angeles (UCLA)
Wild animals	The study did NOT involve wild animals
Field-collected samples	Samples were NOT collected from the field
Ethics oversight	Mice were used under the UCLA Animal Research Committee protocol # 2004-159-43I

Note that full information on the approval of the study protocol must also be provided in the manuscript.

Clinical data

Policy information about clinical studies

All manuscripts should comply with the ICMJEguidelines for publication of clinical research and a completedCONSORT checklist must be included with all submissions.

Clinical trial registration	NCT02869295
Study protocol	*Note where the full trial protocol can be accessed OR if not available, explain why.*
Data collection	*Describe the settings and locales of data collection, noting the time periods of recruitment and data collection.*
Outcomes	*Describe how you pre-defined primary and secondary outcome measures and how you assessed these measures.*
